# A molecular docking study of phytochemical estrogen mimics from dietary herbal supplements

**DOI:** 10.1186/s40203-015-0008-z

**Published:** 2015-03-22

**Authors:** Chelsea N Powers, William N Setzer

**Affiliations:** Department of Chemistry, University of Alabama in Huntsville, Huntsville, AL 35899 USA

**Keywords:** Molecular docking, Estrogen receptor, Herbal supplements

## Abstract

**Purpose:**

The purpose of this study is to use a molecular docking approach to identify potential estrogen mimics or anti-estrogens in phytochemicals found in popular dietary herbal supplements.

**Methods:**

In this study, 568 phytochemicals found in 17 of the most popular herbal supplements sold in the United States were built and docked with two isoforms of the estrogen receptor, ERα and ERβ (a total of 27 different protein crystal structures).

**Results:**

The docking results revealed six strongly docking compounds in *Echinacea*, three from milk thistle (*Silybum marianum*), three from *Gingko bilob*a, one from *Sambucus nigra*, none from maca (*Lepidium meyenii*), five from chaste tree (*Vitex agnus-castus*), two from fenugreek (*Trigonella foenum-graecum*), and two from *Rhodiola rosea*. Notably, of the most popular herbal supplements for women, there were numerous compounds that docked strongly with the estrogen receptor: Licorice (*Glycyrrhiza glabra*) had a total of 26 compounds strongly docking to the estrogen receptor, 15 with wild yam (*Dioscorea villosa*), 11 from black cohosh (*Actaea racemosa*), eight from muira puama (*Ptychopetalum olacoides* or *P. uncinatum*), eight from red clover (*Trifolium pratense*), three from damiana (*Turnera aphrodisiaca* or *T. diffusa*), and three from dong quai (*Angelica sinensis*). Of possible concern were the compounds from men’s herbal supplements that exhibited strong docking to the estrogen receptor: *Gingko biloba* had three compounds, gotu kola (*Centella asiatica*) had two, muira puama (*Ptychopetalum olacoides* or *P. uncinatum*) had eight, and *Tribulus terrestris* had six compounds.

**Conclusions:**

This molecular docking study has revealed that almost all popular herbal supplements contain phytochemical components that may bind to the human estrogen receptor and exhibit selective estrogen receptor modulation. As such, these herbal supplements may cause unwanted side effects related to estrogenic activity.

## Background

**Figure 1:**
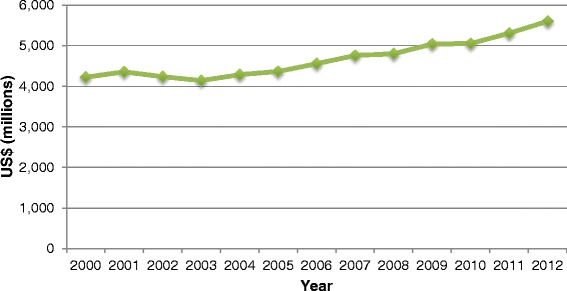
**Relationship between herbal supplement purchases in the United States and the year.**

The use of alternative medicines in the United States, particularly herbal supplements, has dramatically increased since the beginning of the 21st century (Figure 1). Filling American minds with promises of enhanced beauty, sharper senses, and optimum organ functions, herbal supplements claim to increase, or improve almost all issues a person could have with their body. Without a doubt it is appealing to have problems solved by simply swallowing a pill or drinking a tea, not much effort required, however it has been widely ignored the consecutive consequences these supplements can provide (Cupp 1999).

Two major factors play a part in the ongoing, unnoticed herbal supplement crisis: Regulations for herbal supplements and uneducated consumers. Beginning with the first, the United States does not classify herbal supplements as drugs, and therefore supplements are not required to undergo the extensive testing that pharmaceutical drugs do before put on the market. Courtesy of the “Dietary Supplement Health and Education Act of 1994”, herbal supplements are not evaluated by the Food and Drug Administration (Calixto 2000) making it easy for supplement companies to rapidly introduce new supplements to consumers, with or without the knowledge of possible harmful side effects. Unspecified drugs, contaminations, toxins, and/or heavy metals (Au et al. 2000) can be included in an herbal supplement, and since companies are not required to subject their products to quality analysis, this spectrum of harmful compounds could be digested by a consumer and induce adverse effects. As for the second, biologically uneducated consumers do not understand or simply do not consider the concept that plants are not always beneficial. They believe anything that is natural must be good for their health and safe to consume (Stonemetz 2008), which is far from the truth. Plants contain hundreds of phytochemicals, some of which are indeed toxic to the human body. One class of phytochemicals of major concern, which is the focus of this study, phytoestrogens, can interfere and react with the human estrogen receptors, which regulate neural, skeletal, cardiovascular, and reproductive tissues. This interference, however, is not always adverse. For example, some phytoestrogens can promote carcinogenic growth, while others can inhibit the growth.

The purpose of this study was to identify potential estrogen mimics or anti-estrogens in phytochemicals found in popular dietary herbal supplements. The data gathered can only suggest the possibility of a phytochemical to be an anti-estrogen or a mimic, not confirm its estrogenic properties. It is our hope that the discoveries made during this study can help to identify the estrogenic activity of the phytochemicals examined. This information can then lead to the health benefits or hazards associated with the phytochemicals, which in turn could greatly affect the increasingly popular herbal supplement movement.

## Methods

### Literature survey

A literature survey on herbal supplements was carried out to identify the most popular general [*Echinacea*, milk thistle (*Silybum marianum*), *Ginkgo biloba*, *Sambucus nigra*, maca (*Lepidium meyenii*), chaste tree (*Vitex agnus-castus*), fenugreek (*Trigonella foenum-graecum*), and *Rhodiola rosea*], women’s [damiana leaf (*Turnera aphrodisiaca*, *T. diffusa*), muira puama (*Ptychopetalum olacoides*, *P. uncinatum*), black cohosh (*Actaea racemosa* = *Cimifuga racemosa*), licorice root (*Glycyrrhiza glabra*), wild yam (*Dioscorea villosa*), dong quai (*Angelica sinensis*) and red clover (*Trifolium pretense*)], and men’s [*Gingko biloba*, gotu kola (*Centella asiatica*), muira puama (*Ptychopetalum olacoides*, *P. uncinatum*), and *Tribulus terrestris*] herbal supplements advertised and used in the United States. A survey of the literature, including the *Dictionary of Natural Products* (2014) and Duke’s Phytochemical Database (1998), was carried out to determine the phytochemical constituents of each herb.

### Molecular modeling of phytochemicals

Each phytochemical ligand structure (see Figures 2, 3, 4, 5, 6, 7, 8, 9, 10, 11, 12, 13, 14, 15, 16, 17, 18, 19, 20, 21, 22, 23, 24, 25, 26, 27, 28, 29, 30, 31, 32, 33, 34, 35, 36, 37, 38, 39, 40, 41, 42, and 43) was built using Spartan ’14 for Windows (2013). For each ligand, a conformational search and geometry optimization was carried out using the MMFF force field (Halgren 1996).

**Figure 2:**
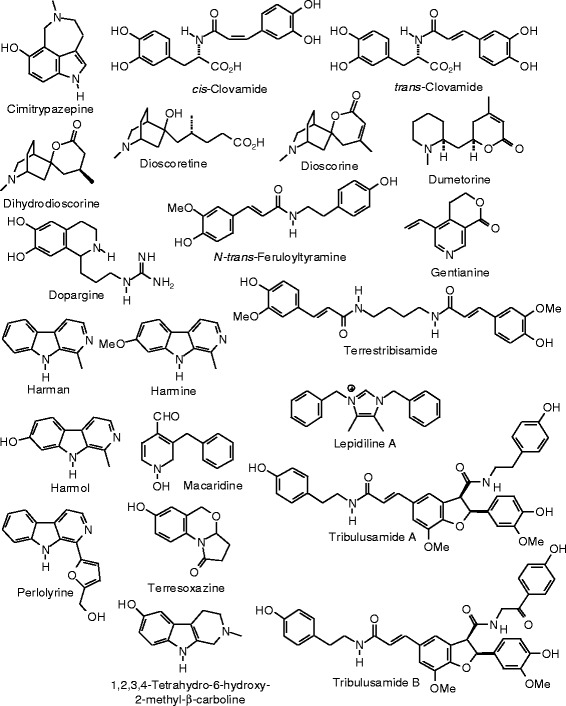
**Alkaloid ligands examined in this work.**

**Figure 3:**
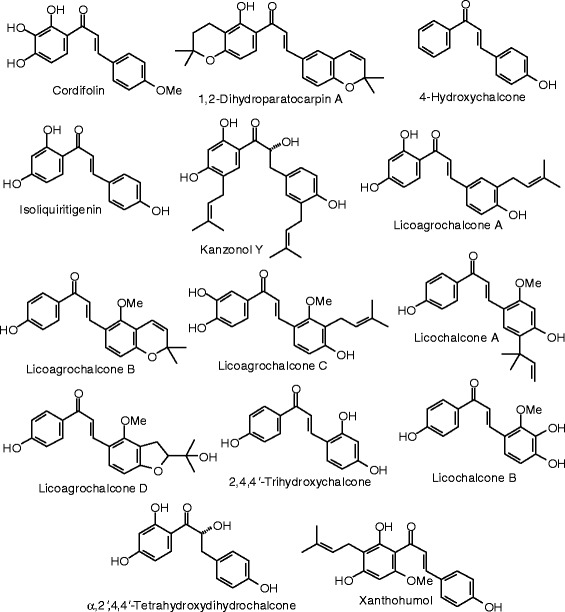
**Chalcone ligands examined in this work.**

**Figure 4:**
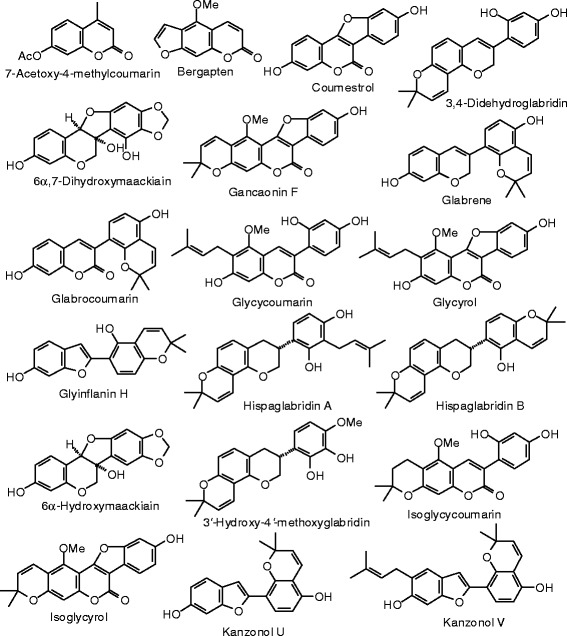
**Coumarin ligands examined in this work.**

**Figure 5:**
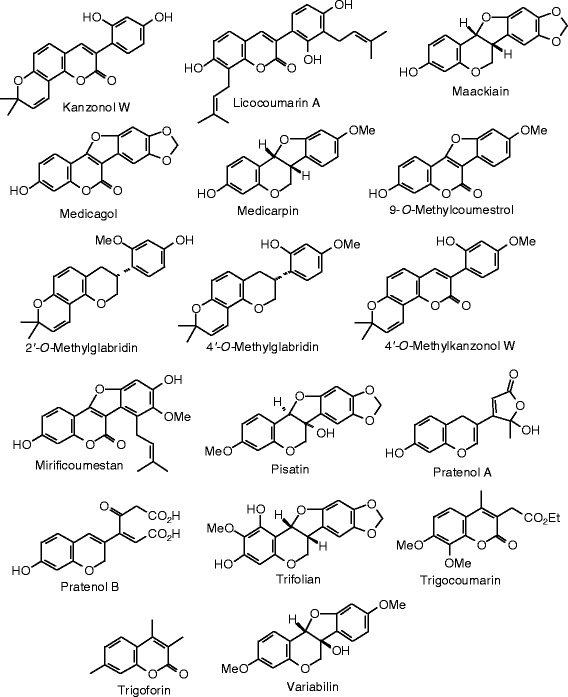
**Additional coumarin ligands examined in this work.**

**Figure 6:**
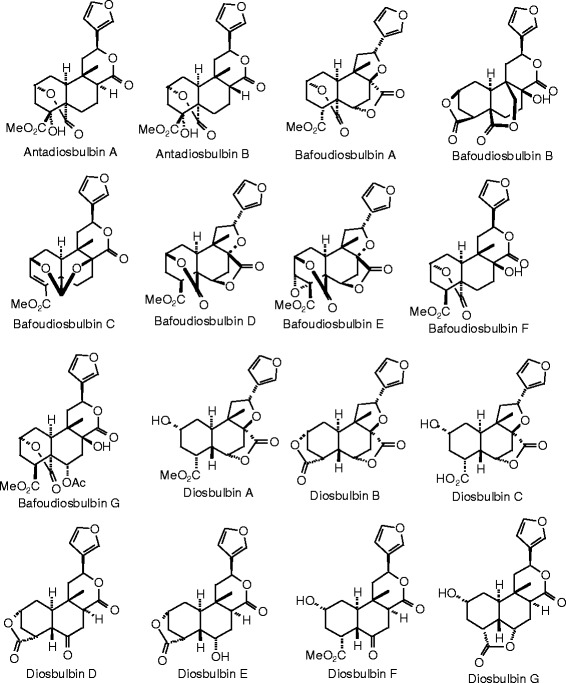
**Diterpenoid ligands examined in this work.**

**Figure 7:**
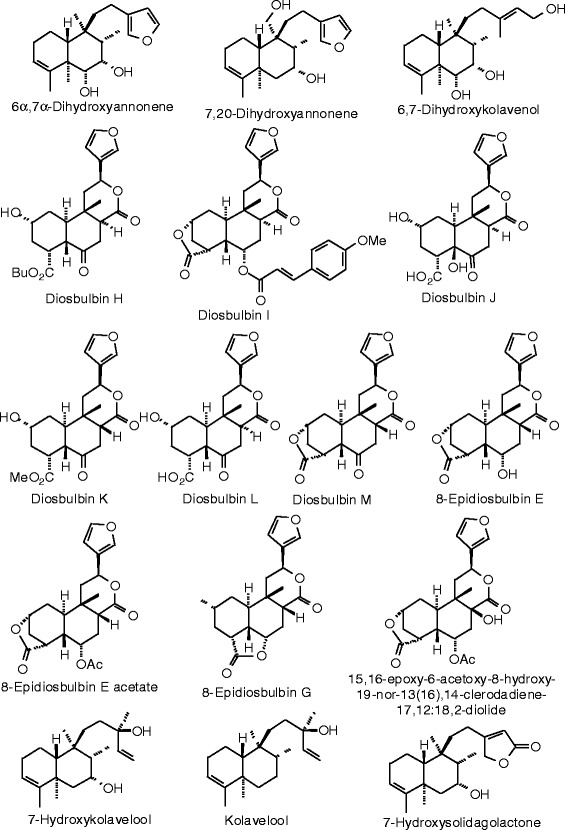
**Additional diterpenoid ligands examined in this work.**

**Figure 8:**
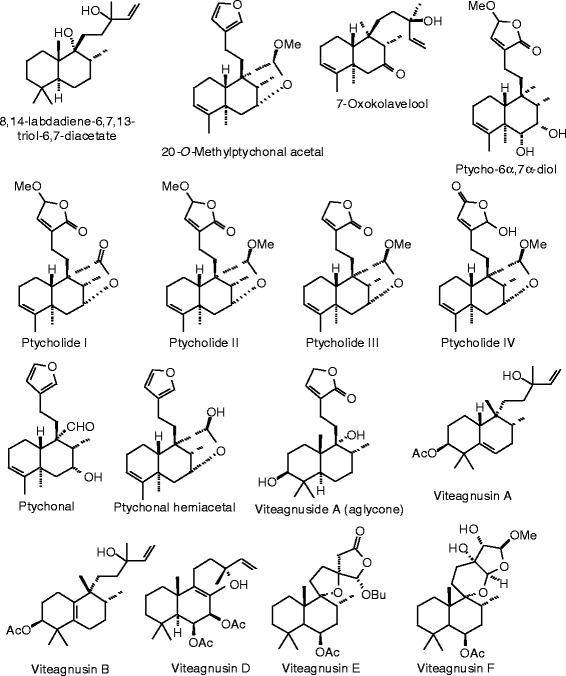
**Additional diterpenoid ligands examined in this work.**

**Figure 9:**
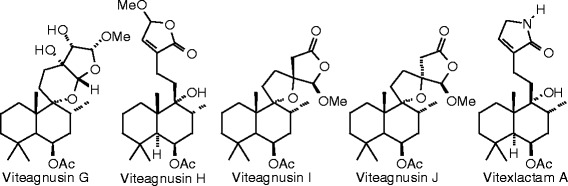
**Additional diterpenoid ligands examined in this work.**

**Figure 10:**
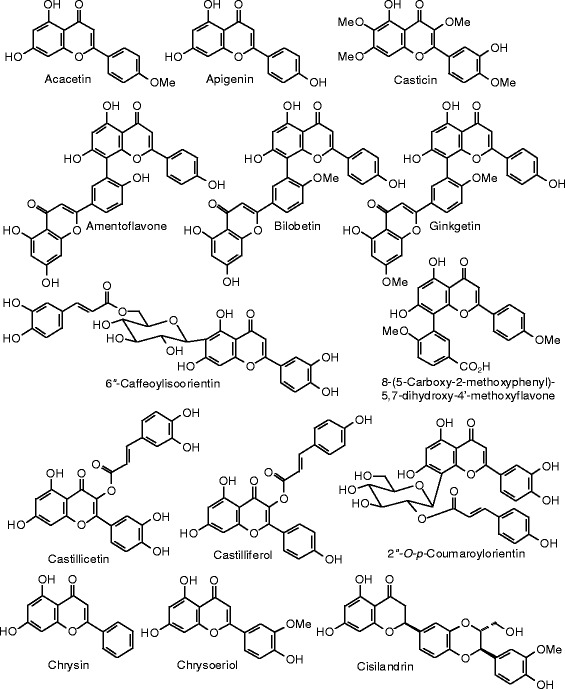
**Flavonoid ligands examined in this work.**

**Figure 11:**
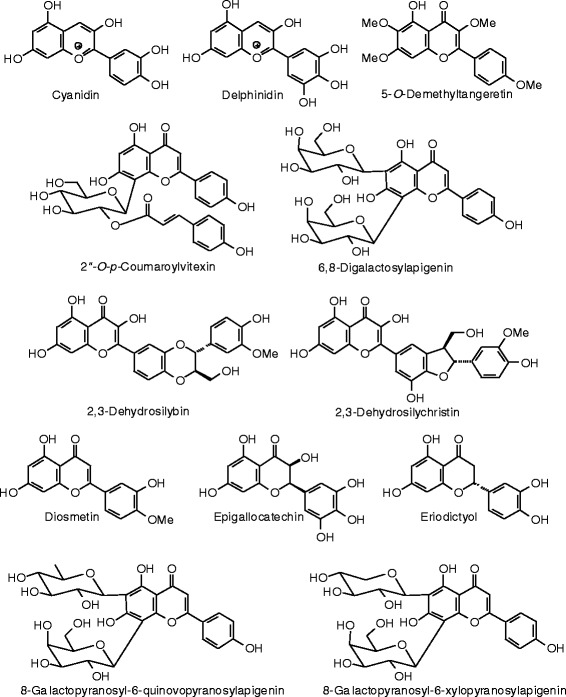
**Additional flavonoid ligands examined in this work.**

**Figure 12:**
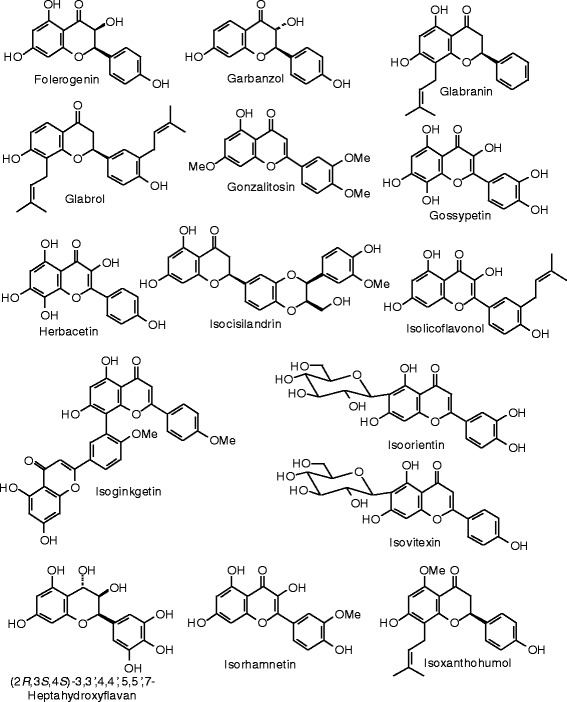
**Additional flavonoid ligands examined in this work.**

**Figure 13:**
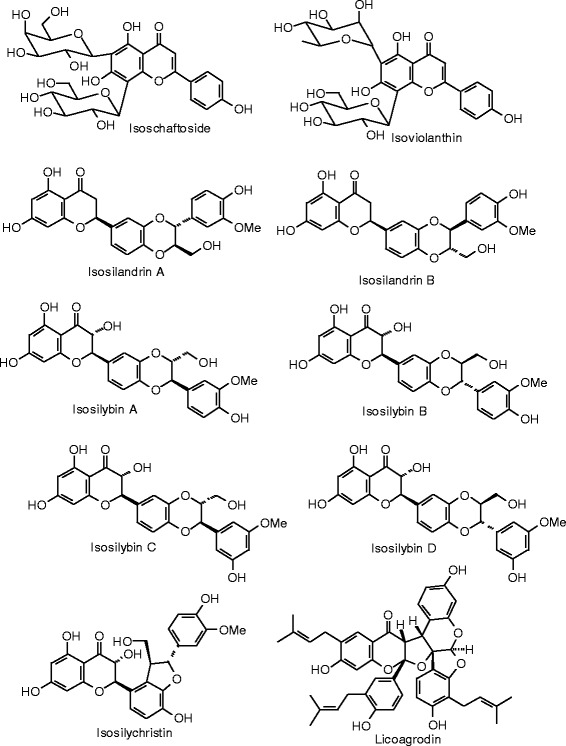
**Additional flavonoid ligands examined in this work.**

**Figure 14:**
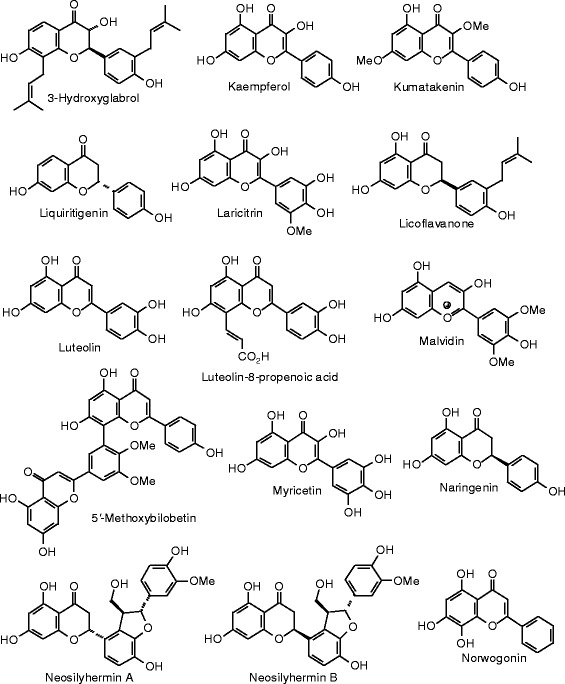
**Additional flavonoid ligands examined in this work.**

**Figure 15:**
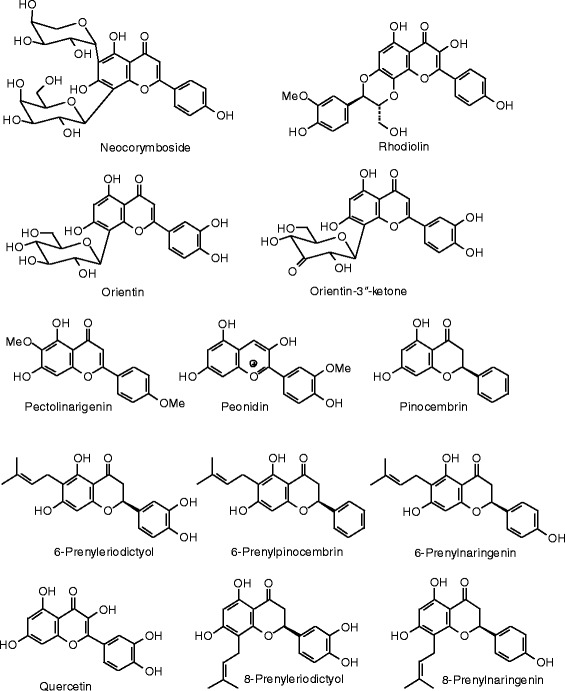
**Additional flavonoid ligands examined in this work.**

**Figure 16:**
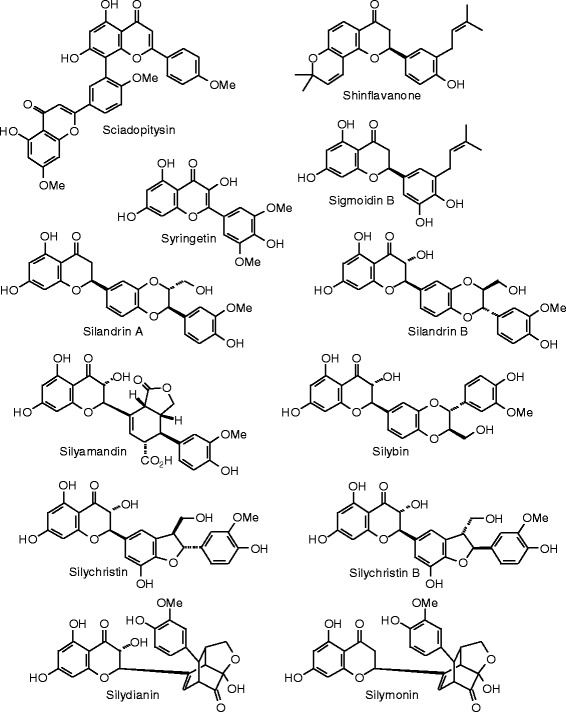
**Additional flavonoid ligands examined in this work.**

**Figure 17:**
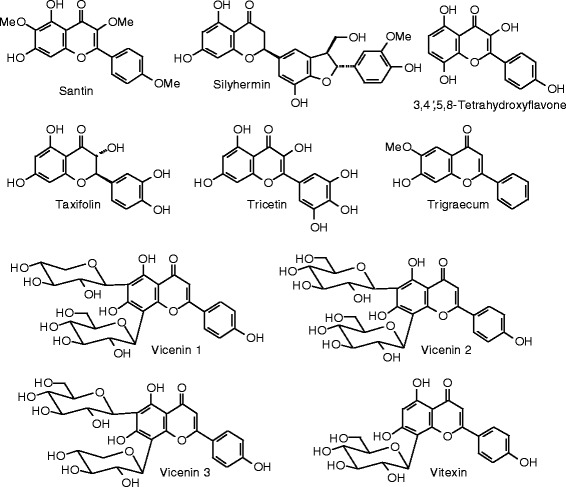
**Additional flavonoid ligands examined in this work.**

**Figure 18:**
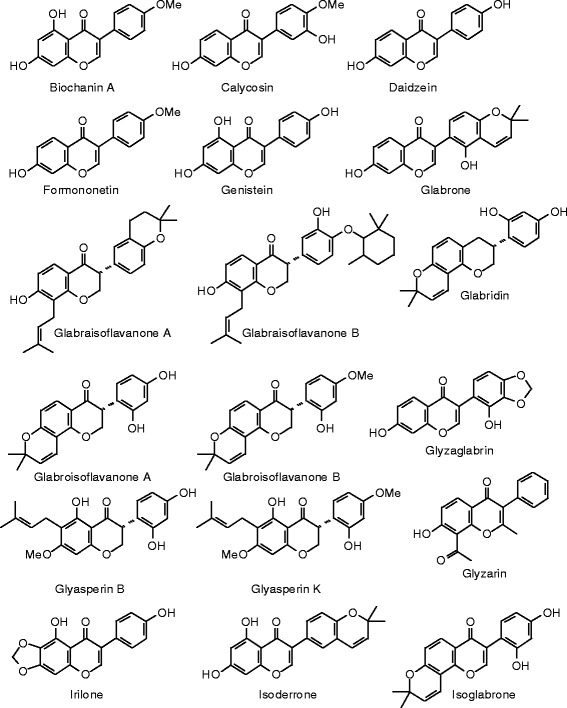
**Isoflavonoid ligands examined in this work.**

**Figure 19:**
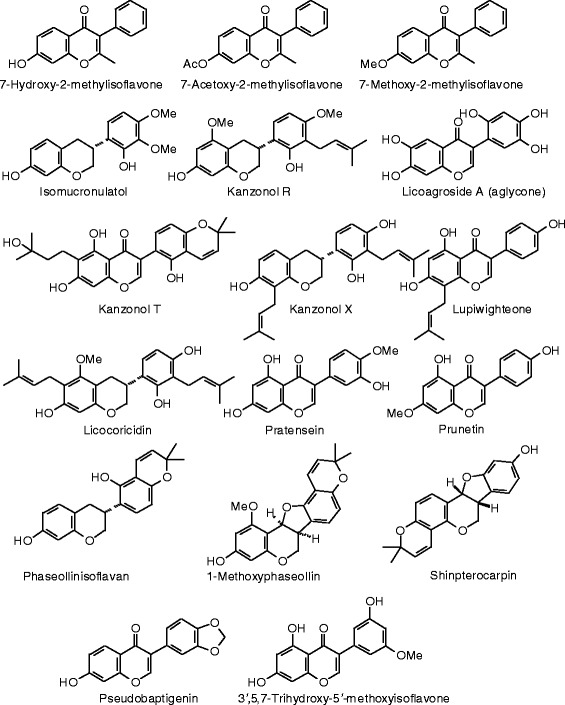
**Additional isoflavonoid ligands examined in this work.**

**Figure 20:**
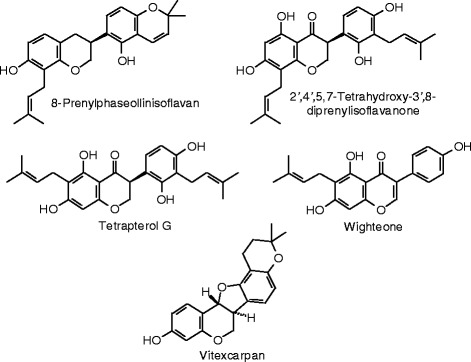
**Additional isoflavonoid ligands examined in this work.**

**Figure 21:**
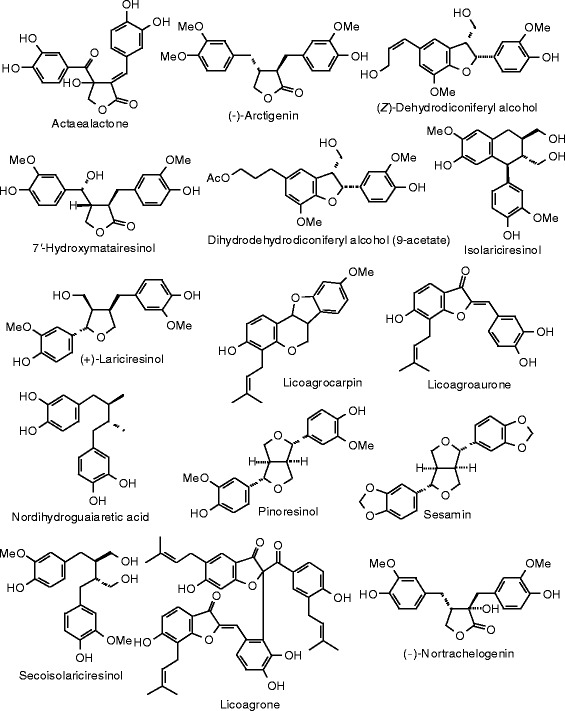
**Lignan ligands examined in this work.**

**Figure 22:**
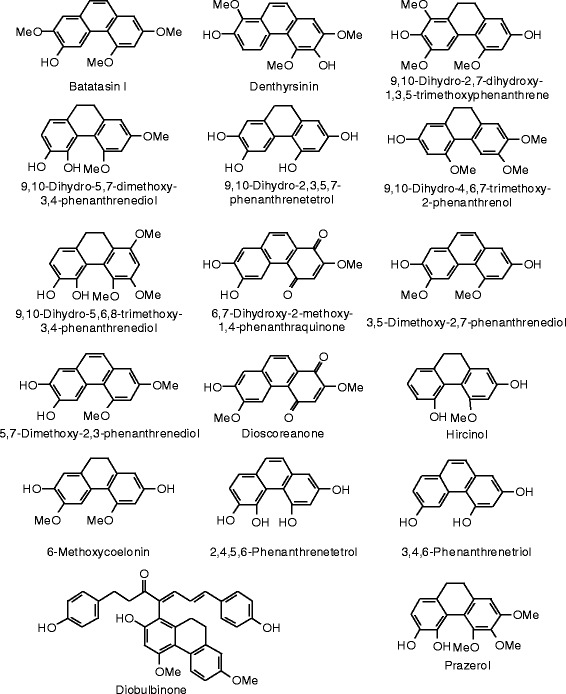
**Phenanthrenoid ligands examined in this work.**

**Figure 23:**
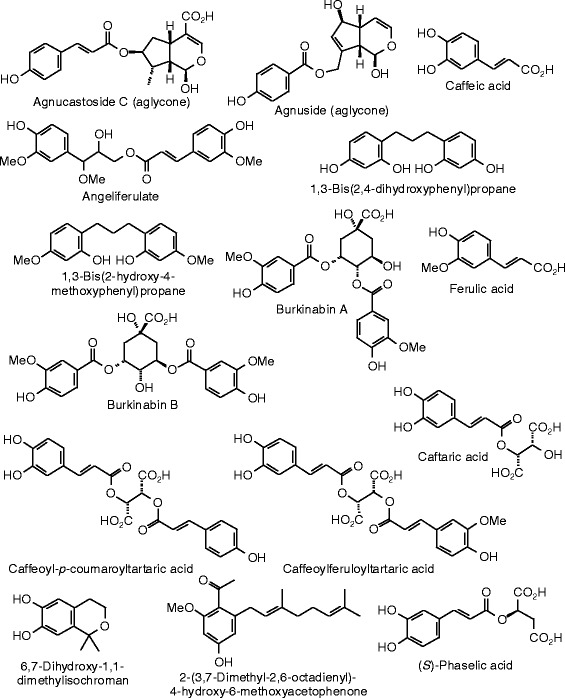
**Miscellaneous phenolic compounds examined in this work.**

**Figure 24:**
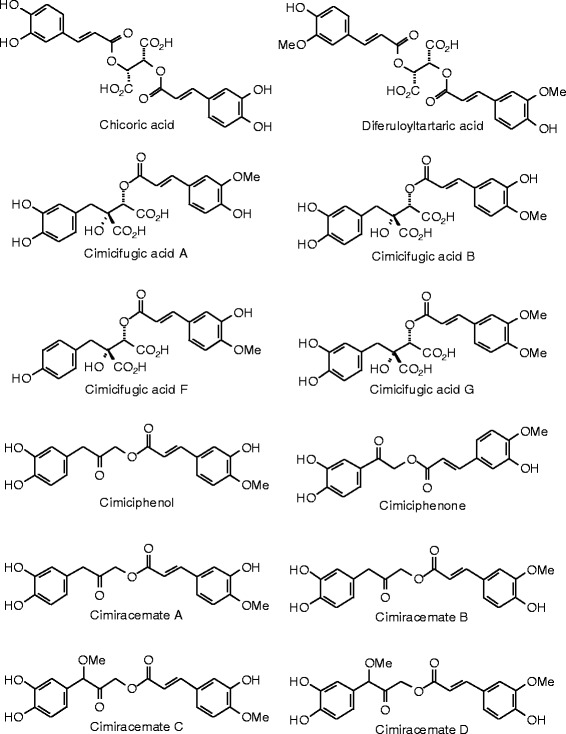
**Additional miscellaneous phenolic compounds examined in this work.**

**Figure 25:**
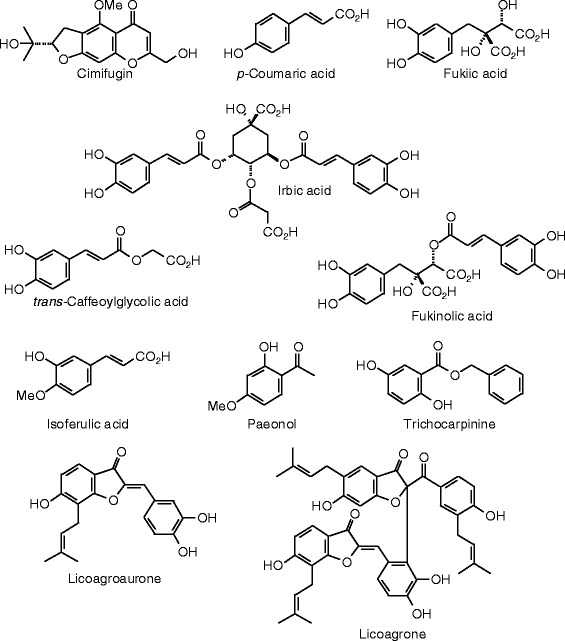
**Additional miscellaneous phenolic compounds examined in this work.**

**Figure 26:**
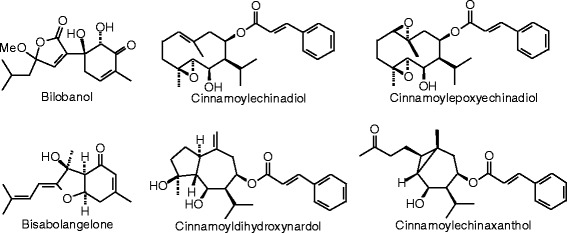
**Sesquiterpenoid ligands examined in this work.**

**Figure 27:**
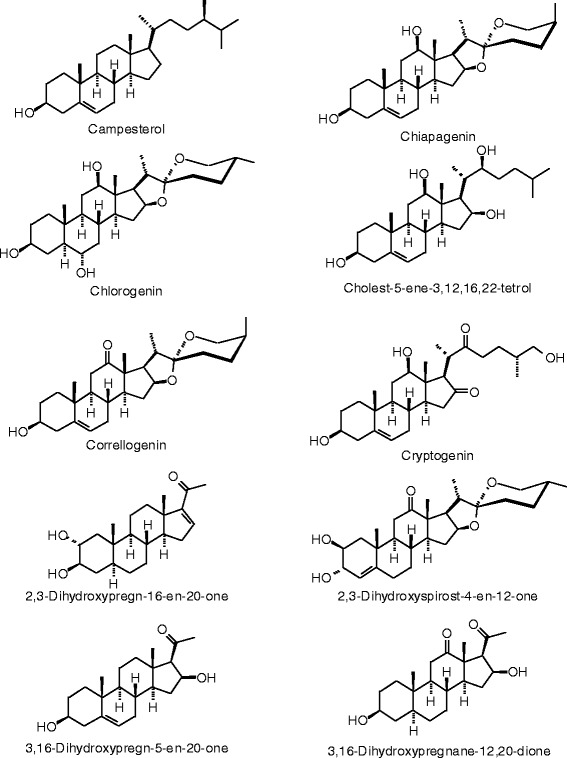
**Steroid ligands examined in this work.**

**Figure 28:**
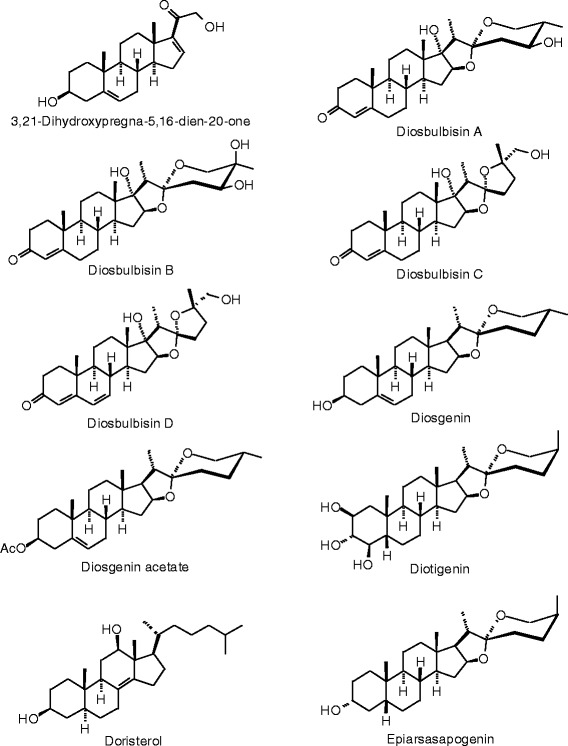
**Additional steroid ligands examined in this work.**

**Figure 29:**
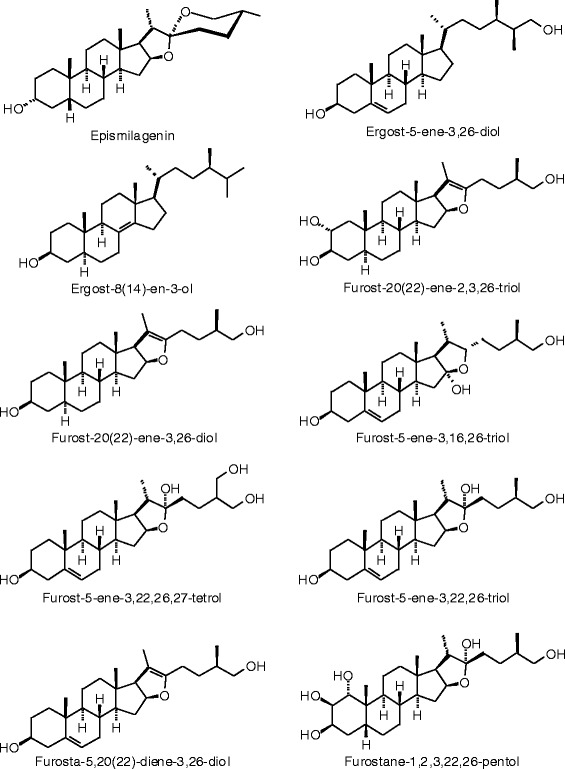
**Additional steroid ligands examined in this work.**

**Figure 30:**
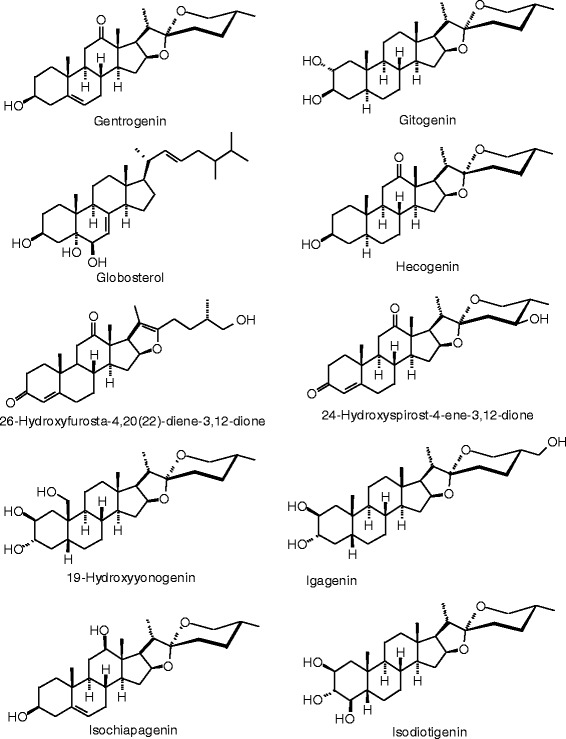
**Additional steroid ligands examined in this work.**

**Figure 31:**
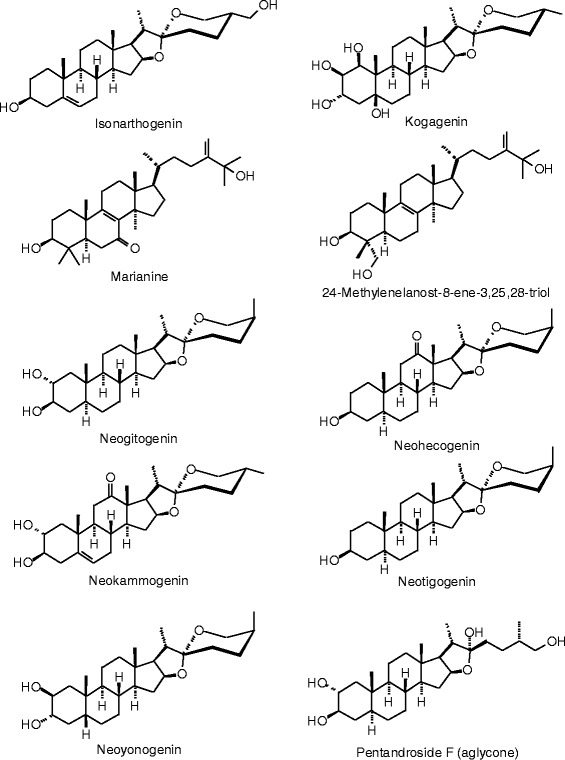
**Additional steroid ligands examined in this work.**

**Figure 32:**
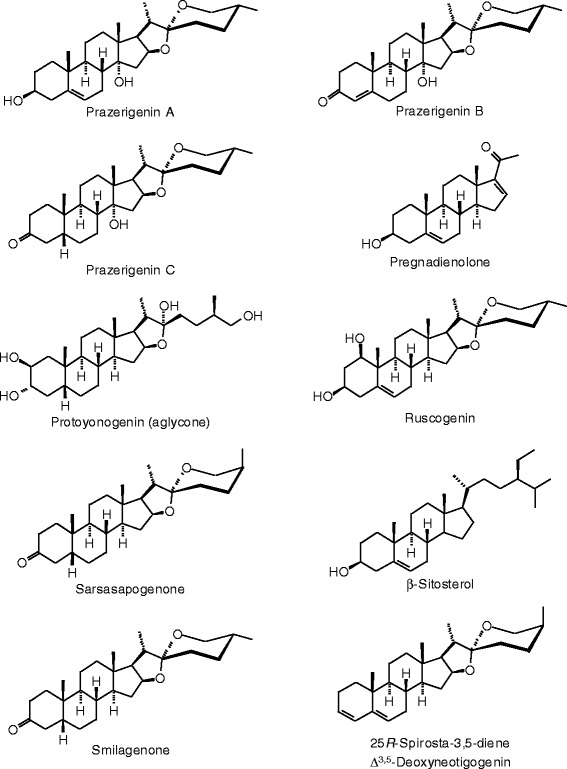
**Additional steroid ligands examined in this work.**

**Figure 33:**
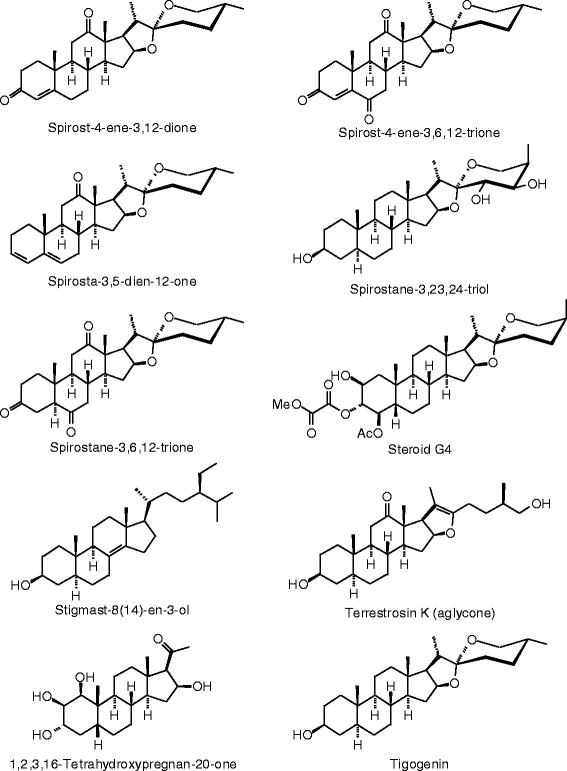
**Additional steroid ligands examined in this work.**

**Figure 34:**
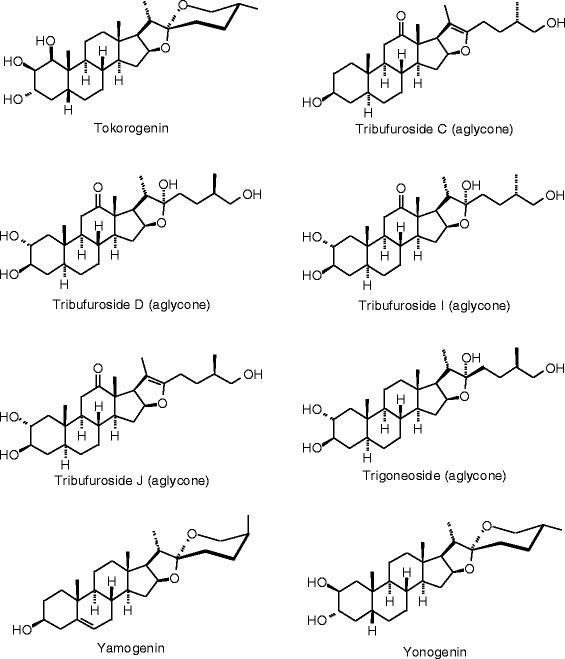
**Additional steroid ligands examined in this work.**

**Figure 35:**
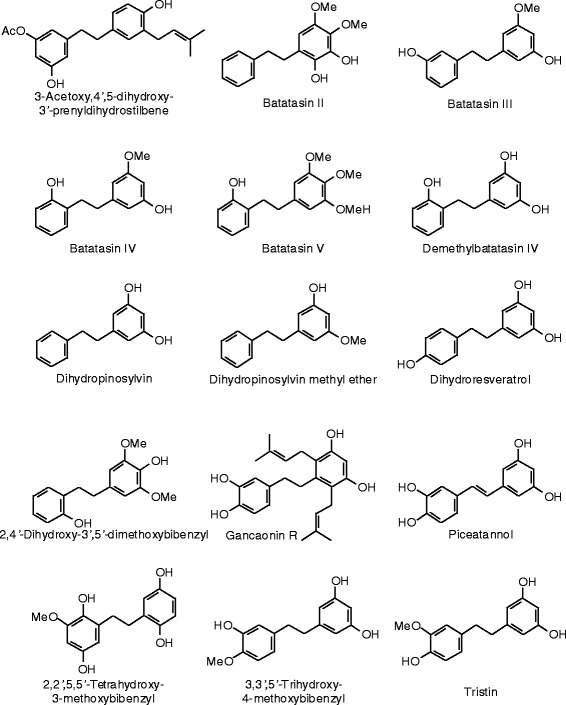
**Stilbenoid ligands examined in this work.**

**Figure 36:**
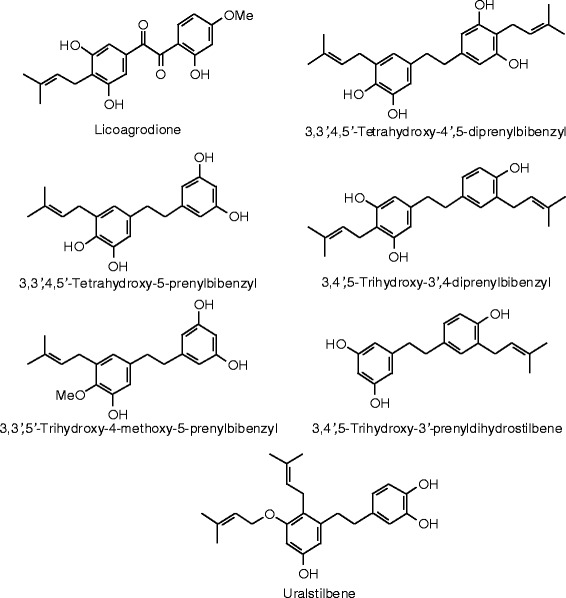
**Additional stilbenoid ligands examined in this work.**

**Figure 37:**
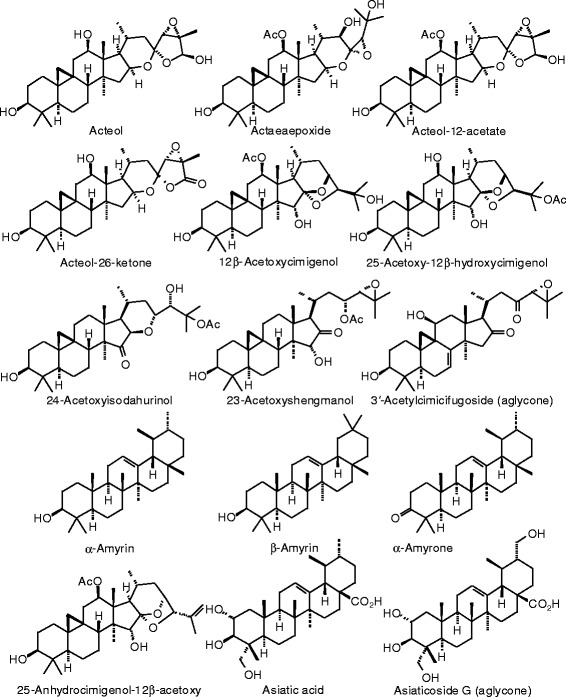
**Triterpenoid ligands examined in this work.**

**Figure 38:**
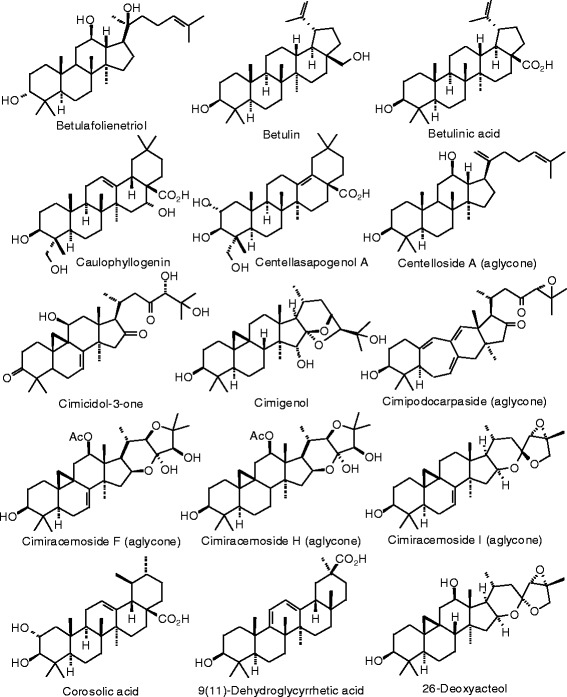
**Additional triterpenoid ligands examined in this work.**

**Figure 39:**
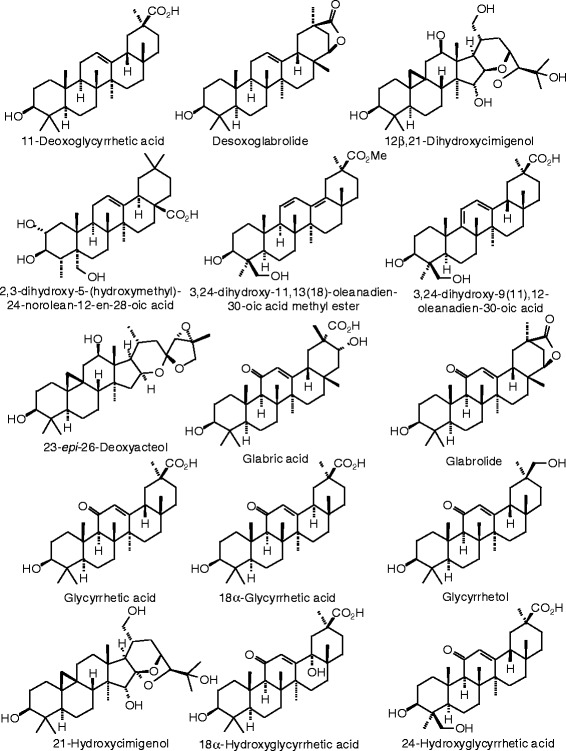
**Additional triterpenoid ligands examined in this work.**

**Figure 40:**
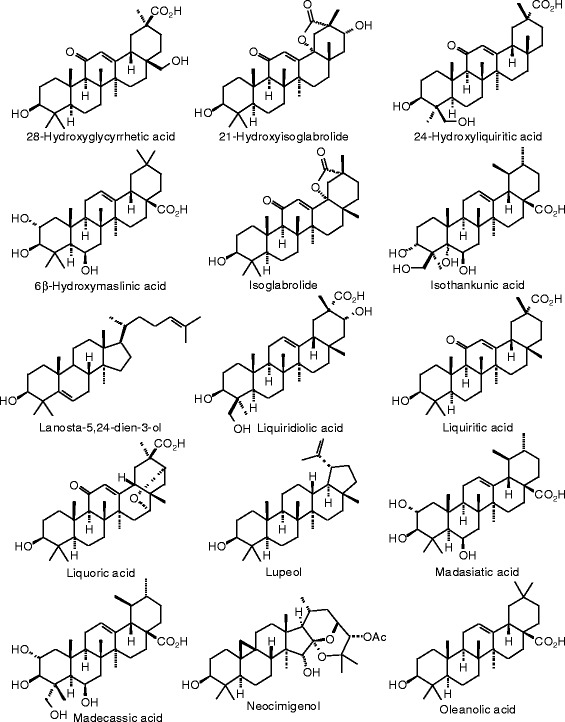
**Additional triterpenoid ligands examined in this work.**

**Figure 41:**
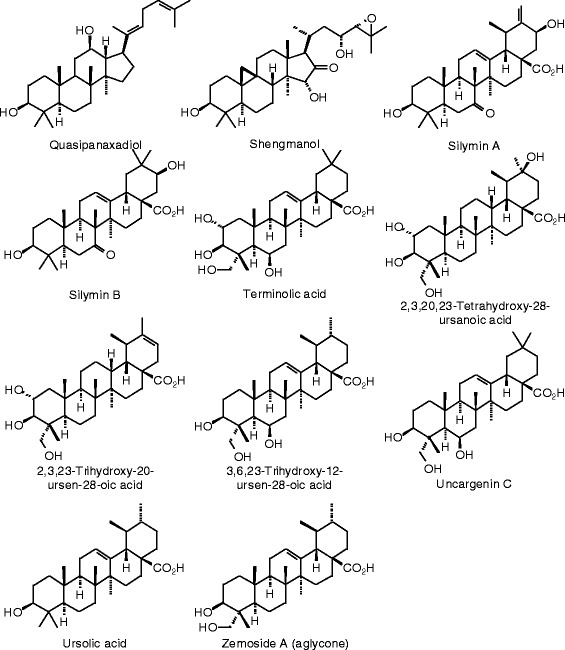
**Additional triterpenoid ligands examined in this work.**

**Figure 42:**
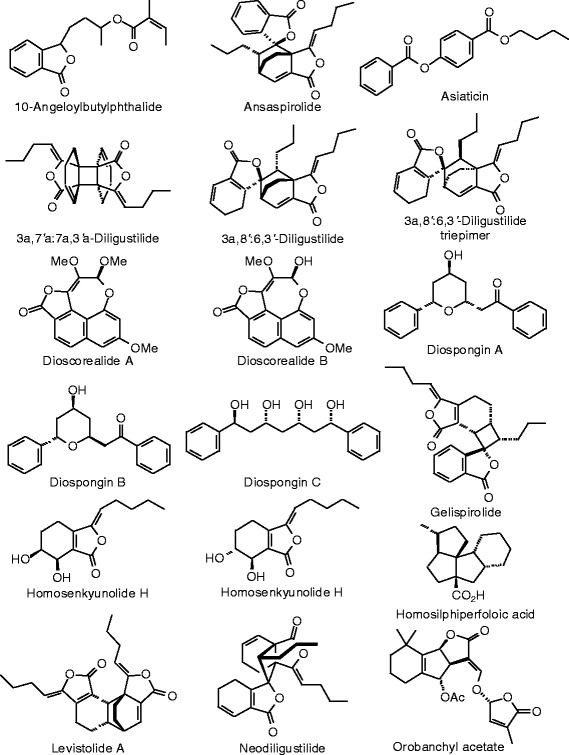
**Miscellaneous phytochemical ligands examined in this work.**

**Figure 43:**
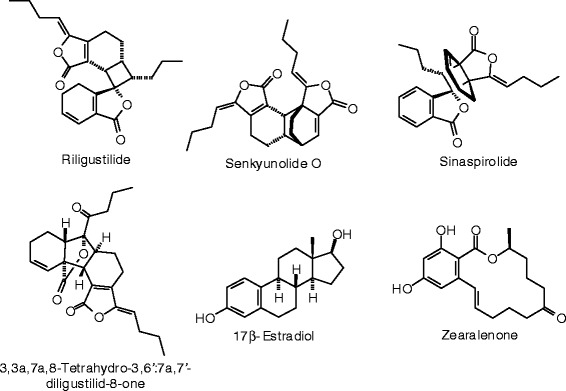
**Additional miscellaneous phytochemical ligands examined in this work.**

### Molecular docking

Protein-ligand docking studies were carried out based on the crystal structures of human estrogen receptor α [ERα: PDB 1X7E (Manas et al. 2004a), PDB 1X7R (Manas et al. 2004b), and PDB 3ERD (Shiau et al. 1998)] and human estrogen receptor β [ERβ: PDB 1U3Q, 1U3R, 1U3S (Malamas et al. 2004), 1U9E, 1X7B, 1X76, 1X78 (Manas et al. 2004a), and 1X7J (Manas et al. 2004b)]. Prior to docking all solvent molecules and the co-crystallized ligands were removed from the structures. Molecular docking calculations for all compounds with each of the proteins were undertaken using Molegro Virtual Docker v. 6.0 (2013). Potential binding sites in the protein structures were identified using the grid-based cavity prediction algorithm of the Molegro Virtual Docker (2013) program. The location of the volume used by the docking search algorithm was positioned at the center of the cavity and a sphere (15 Å radius) large enough to encompass the entire cavity of the binding site of each protein structure was selected in order to allow each ligand to search. If a co-crystallized inhibitor or substrate was present in the structure, then that site was chosen as the binding site. If no co-crystallized ligand was present, then suitably sized (>50 Å^3^) cavities were used as potential binding sites. The docking searches were constrained to those cavities. Standard protonation states of the proteins based on neutral pH were used in the docking studies. Each protein was used as a rigid model structure; no relaxation of the protein was performed. Assignments of charges on each protein were based on standard templates as part of the Molegro Virtual Docker (2013) program (Thomsen and Christensen 2006); no other charges were necessary to be set. Flexible ligand models were used in the docking and subsequent optimization scheme. As a test of docking accuracy and for docking energy comparison, co-crystallized ligands were re-docked into the protein structures (see Table 1). Additionally, as positive controls, the known estrogenic compounds 17β-estradiol and α-zearalenone were docked with each protein structure in order to compare docking energies with the herbal phytochemicals. Different orientations of the ligands were searched and ranked based on their energy scores. The RMSD threshold for multiple cluster poses was set at <1.00 Å. The docking algorithm was set at maximum iterations of 1500 with a simplex evolution population size of 50 and a minimum of 30 runs for each ligand. Each binding site of oligomeric structures was searched with each ligand. The lowest-energy (strongest-docking) poses for each ligand in each protein target are summarized in Tables 2, 3, 4, 5, 6, 7, 8, 9, 10, 11, 12, 13, 14 and 15.

**Table 1:** **MolDock docking energies of co-crystallized ligands and root-mean-squared deviations between the co-crystallized ligand and the re-docked poses of the co-crystallized ligand with human estrogen receptors α and β**

**Protein**	**PDB code**	**Co-crystallized ligand**	***E***_**dock**_ **(kJ/mol)**	**RMSD (Å)**
ERα	1X7E	[5-hydroxy-2-(4-hydroxyphenyl)-1-benzofuran-7-yx]acetonitrile	−100.9	0.46
	1X7R	genistein	−95.3	0.44
	3ERD	diethylstilbestrol	−97.0	0.75
ERβ	1U3Q	4-(6-hydroxybenzo[d]isoxazol-3-yl)benzene-1,3-diol	−98.9	1.40
	1U3R	2-(5-hydroxynaphthalen-1-yl)-1,3-benzooxazol-6-ol	−111.3	0.36
	1U3S	3-(6-hydroxynaphthalen-2-yl)-benzo[d]isoxazol-6-ol	−107.7	0.35
	1U9E	2-(4-hydroxyphenyl)benzofuran-5-ol	−90.4	0.62
	1X7B	2-(3-fluoro-4-hydroxyphenyl)-7-vinyl-1,3-benzoxazol-5-ol	−107.9	0.46
	1X7J	genistein	−99.9	0.66
	1X76	5-hydroxy-2-(4-hydroxyphenyl)-1-benzofuran-7-carbonitrile	−101.3	0.42
	1X78	[5-hydroxy-2-(4-hydroxyphenyl)-1-benzofuran-7-yl]carbonitrile	−107.7	0.40

**Table 2:** **MolDock molecular docking energies (kJ/mol) for alkaloids with human estrogen receptors α and β**

**Compound**	**Plant Source**	**ERα**	**ERβ**
cimitrypazepine	*Cimicifuga racemosa*	−87.6	−75.5
*cis-*clovamide	*Trifolium pratense*	−119.8	−124.9
*trans-*clovamide	*Trifolium pratense*	−113.6	−122.0
dihydrodioscorine	*Dioscorea* spp.	−60.2	−64.9
dioscoretine	*Dioscorea* spp.	−81.8	−80.9
dioscorine	*Dioscorea* spp.	−62.1	−68.1
dopargine	*Cimicifuga racemosa*	−97.9	−100.6
dumetorine	*Dioscorea* spp.	−77.0	−82.3
*N-trans-*feruloyltyramine	*Tribulus terrestris*	−103.1	−113.8
gentianine	*Trigonella foenum-graecum*	−67.2	−64.9
harman	*Tribulus terrestris*	−74.9	−67.4
harmine	*Tribulus terrestris*	−68.8	−78.0
harmol	*Tribulus terrestris*	−85.8	−75.5
lepidiline A	*Lepidium meyenii*	−91.2	−96.9
macaridine	*Lepidium meyenii*	−78.0	−78.2
perlolyrine	*Tribulus terrestris*	−93.4	−104.6
terresoxazine	*Tribulus terrestris*	−66.3	−74.7
terrestribisamide	*Tribulus terrestris*	−102.1	−101.2
1,2,3,4-tetrahydro-6-hydroxy-2-methyl-β-carboline	*Cimicifuga racemosa*	−73.5	−78.2
Tribulusamide A	*Tribulus terrestris*	−48.7	no dock
Tribulusamide B	*Tribulus terrestris*	no dock	no dock

**Table 3:** **MolDock molecular docking energies (kJ/mol) for chalcones with human estrogen receptors α and β**

**Compound**	**Plant Source**	**ERα**	**ERβ**
cordifolin	*Glycyrrhiza glabra*	−102.2	−110.2
1,2-dihydroparatocarpin A	*Glycyrrhiza glabra*	no dock	−11.2
4-hydroxychalcone	*Glycyrrhiza glabra*	−88.4	−94.5
isoliquiritigenin	*Glycyrrhiza glabra*	−99.9	−102.6
kanzonol Y	*Glycyrrhiza glabra*	−111.2	−122.4
licoagrochalcone A	*Glycyrrhiza glabra*	−102.4	−115.5
licoagrochalcone B	*Glycyrrhiza glabra*	−55.8	−112.0
licoagrochalcone C	*Glycyrrhiza glabra*	−90.7	−103.1
licoagrochalcone D	*Glycyrrhiza glabra*	−14.6	−100.5
licochalcone A	*Glycyrrhiza glabra*	−93.2	−107.8
licochalcone B	*Glycyrrhiza glabra*	−107.8	−108.9
α,2′,4,4′-tetrahydroxydihydrochalcone	*Glycyrrhiza glabra*	−98.3	−105.0
2,4,4′-trihydroxychalcone	*Glycyrrhiza glabra*	−103.4	−104.9
xanthohumol	*Glycyrrhiza glabra*	−116.8	−116.8

**Table 4:** **MolDock molecular docking energies (kJ/mol) for coumarins with human estrogen receptors α and β**

**Compound**	**Plant Source**	**ERα**	**ERβ**
7-acetoxy-4-methylcoumarin	*Trigonella foenum-graecum*	−74.6	−77.6
bergapten	*Glycyrrhiza glabra*	−71.2	−77.6
coumestrol	*Glycyrrhiza glabra*	−89.4	−99.8
	*Trifolium pratense*		
3,4-didehydroglabridin	*Glycyrrhiza glabra*	−34.7	−90.0
6α,7-dihydroxymaackiain	*Trifolium pratense*	−97.7	−107.9
gancaonin F	*Glycyrrhiza glabra*	−37.5	−109.6
glabrene	*Glycyrrhiza glabra*	−104.8	−114.9
glabrocoumarin	*Glycyrrhiza glabra*	−99.0	−109.7
glycycoumarin	*Glycyrrhiza glabra*	−75.2	−110.2
glycyrol	*Glycyrrhiza glabra*	−49.2	−108.8
glyinflanin H	*Glycyrrhiza glabra*	−53.3	−102.1
hispaglabridin A	*Glycyrrhiza glabra*	−68.5	−84.3
hispaglabridin B	*Glycyrrhiza glabra*	−42.8	−59.3
6α-hydroxymaackiain	*Trifolium pratense*	−95.9	−102.9
3′-hydroxy-4′-methoxyglabridin	*Glycyrrhiza glabra*	−35.9	−86.9
isoglycycoumarin	*Glycyrrhiza glabra*	−20.3	−77.6
isoglycyrol	*Glycyrrhiza glabra*	−10.8	−66.1
kanzonol U	*Glycyrrhiza glabra*	−102.8	−109.9
kanzonol V	*Glycyrrhiza glabra*	−23.7	−71.3
kanzonol W	*Glycyrrhiza glabra*	−20.4	−82.5
licocoumarin A	*Glycyrrhiza glabra*	−54.9	−24.2
maackiain	*Trifolium pratense*	−84.3	−83.6
medicagol	*Trifolium pratense*	−91.6	−107.6
medicarpin	*Trifolium pratense*	−83.7	−79.1
9-*O*-methylcoumestrol	*Trifolium pratense*	−88.2	−100.8
2′-*O*-methylglabridin	*Glycyrrhiza glabra*	−78.7	−78.2
4′-*O*-methylgrabridin	*Glycyrrhiza glabra*	−25.4	−80.8
4′-*O*-methylkanzonol W	*Glycyrrhiza glabra*	no dock	−69.4
mirificoumestan	*Pueraria mirifica*	−98.9	−113.0
pisatin	*Trifolium pratense*	−84.7	−96.8
pratenol A	*Trifolium pratense*	−93.7	−99.4
pratenol B	*Trifolium pratense*	−104.8	−112.0
trifolian	*Trifolium pratense*	−82.0	−71.9
trigocoumarin	*Trigonella foenum-graecum*	−93.6	−98.1
trigoforin	*Trigonella foenum-graecum*	−59.0	−66.2
variabilin	*Trifolium pratense*	−86.5	−86.9

**Table 5:** **MolDock molecular docking energies (kJ/mol) for diterpenoids with human estrogen receptors α and β**

**Compound**	**Plant Source**	**ERα**	**ERβ**
antadiosbulbin A	*Dioscorea* spp.	−102.7	−68.7
antadiosbulbin B	*Dioscorea* spp.	−76.0	−64.9
bafoudiosbulbin A	*Dioscorea* spp.	−102.2	−79.3
bafoudiosbulbin B	*Dioscorea* spp.	−95.6	−98.9
bafoudiosbulbin C	*Dioscorea* spp.	−68.0	−93.1
bafoudiosbulbin D	*Dioscorea* spp.	−79.3	−75.8
bafoudiosbulbin E	*Dioscorea* spp.	−84.1	−62.2
bafoudiosbulbin F	*Dioscorea* spp.	−86.0	−73.6
bafoudiosbulbin_G	*Dioscorea* spp.	−56.3	−13.0
6α,7α-dihydroxyannonene	*Ptychopetalum olacoides, P. uncinatum*	−95.2	−107.3
7,20-dihydroxyannonene	*Ptychopetalum olacoides, P. uncinatum*	−91.8	−105.5
6,7-dihydroxykolavenol	*Ptychopetalum olacoides, P. uncinatum*	−94.0	−107.0
diosbulbin A	*Dioscorea* spp.	−82.3	−47.7
diosbulbin B	*Dioscorea* spp.	−86.7	−81.8
diosbulbin C	*Dioscorea* spp.	−83.9	−50.1
diosbulbin D	*Dioscorea* spp.	−107.1	−110.4
diosbulbin E	*Dioscorea* spp.	−92.2	−108.1
diosbulbin F	*Dioscorea* spp.	−111.2	−114.8
diosbulbin G	*Dioscorea* spp.	−89.9	−72.0
diosbulbin H	*Dioscorea* spp.	−97.5	−114.1
diosbulbin I	*Dioscorea* spp.	no dock	no dock
diosbulbin J	*Dioscorea* spp.	−106.4	−107.1
diosbulbin K	*Dioscorea* spp.	−112.1	−108.3
diosbulbin L	*Dioscorea* spp.	−110.8	−110.9
diosbulbin M	*Dioscorea* spp.	−103.1	−107.8
8-epidiosbulbin E	*Dioscorea* spp.	−78.3	−72.8
8-epidiosbulbin E acetate	*Dioscorea* spp.	−16.8	−35.0
8-epidiosbulbin G	*Dioscorea* spp.	−89.3	−72.8
15,16-epoxy-6,8-dihydroxy-19-nor-13(16),14-clerodadiene-17,12:18,2-diolide-6-acetate	*Dioscorea* spp.	−38.1	−26.8
7-hydroxykolavelool	*Ptychopetalum olacoides, P. uncinatum*	−90.7	−100.1
7-hydroxysolidagolactone	*Ptychopetalum olacoides, P. uncinatum*	−96.1	−109.4
kolavelool	*Ptychopetalum olacoides, P. uncinatum*	−89.4	−94.8
8,14-labdadiene-6,7,13-triol-6,7-diacetate	*Vitex agnus-castus*	−92.4	−90.7
20-*O*-methylptychonal acetal	*Ptychopetalum olacoides, P. uncinatum*	−92.6	−105.1
7-oxokolavelool	*Ptychopetalum olacoides, P. uncinatum*	−91.6	−99.7
ptycho-6α,7α-diol	*Ptychopetalum olacoides, P. uncinatum*	−103.6	−122.9
ptycholide I	*Ptychopetalum olacoides, P. uncinatum*	−101.0	−108.9
ptycholide II	*Ptychopetalum olacoides, P. uncinatum*	−105.1	−104.6
ptycholide III	*Ptychopetalum olacoides, P. uncinatum*	−98.6	−109.1
ptycholide IV	*Ptychopetalum olacoides, P. uncinatum*	−103.7	−114.7
ptychonal	*Ptychopetalum olacoides, P. uncinatum*	−93.8	−104.7
ptychonal (hemiacetal)	*Ptychopetalum olacoides, P. uncinatum*	−93.4	−105.9
ptychonolide	*Ptychopetalum olacoides, P. uncinatum*	−87.7	−99.9
viteagnuside A (aglycone)	*Vitex agnus-castus*	−100.1	−101.9
viteagnusin A	*Vitex agnus-castus*	−87.4	−94.6
viteagnusin_B	*Vitex agnus-castus*	−75.5	−95.6
viteagnusin D	*Vitex agnus-castus*	−92.4	−97.3
viteagnusin E	*Vitex agnus-castus*	−3.7	−37.0
viteagnusin F	*Vitex agnus-castus*	−7.1	no dock
viteagnusin G	*Vitex agnus-castus*	−44.3	−76.4
viteagnusin H	*Vitex agnus-castus*	−93.5	−94.3
viteagnusin I	*Vitex agnus-castus*	−44.2	−46.1
viteagnusin J	*Vitex agnus-castus*	−54.5	−93.5
vitexlactam A	*Vitex agnus-castus*	−102.4	−99.1

**Table 6:** **MolDock molecular docking energies (kJ/mol) for flavonoids with human estrogen receptors α and β**

**Compound**	**Plant Source**	**ERα**	**ERβ**
acacetin	*Ginkgo biloba*	−83.6	−96.0
	*Turnera aphrodisiaca*		
	*Turnera diffusa*		
amentoflavone	*Ginkgo biloba*	no dock	no dock
apigenin	*Ginkgo biloba*	−88.6	−97.3
	*Silybum marianum*		
	*Turnera aphrodisiaca*		
	*Turnera diffusa*		
	*Vitex agnus-castus*		
bilobetin	*Ginkgo biloba*	no dock	no dock
6*"*-caffeoylisoorientin	*Vitex agnus-castus*	no dock	no dock
6*"*-caffeoylisoorientin(4*"*-methylether)	*Vitex agnus-castus*	no dock	no dock
8-(5-carboxy-2-methoxyphenyl)-5,7-dihydroxy-4*"*-methoxyflavone	*Ginkgo biloba*	−50.1	−78.2
casticin	*Centella asiatica*	−15.6	−106.4
	*Vitex agnus-castus*		
castillicetin	*Centella asiatica*	−59.1	−89.5
castilliferol	*Centella asiatica*	−48.5	−85.2
chrysin	*Ginkgo biloba*	−81.6	−88.2
chrysoeriol	*Silybum marianum*	−94.6	−102.6
cisilandrin	*Silybum marianum*	no dock	no dock
2*"*-*O-p-*coumaroylorientin	*Trigonella foenum-gracum*	no dock	no dock
2*"-O-p-*coumaroylvitexin	*Trigonella foenum-gracum*	no dock	no dock
cyanidin	*Ginkgo biloba*	−89.1	−101.9
	*Trifolium pratense*		
2,3-dehydrosilybin	*Silybum marianum*	no dock	no dock
2,3-dehydrosilychristin	*Silybum marianum*	no dock	no dock
delphinidin	*Trifolium pratense*	−91.6	−102.3
5-*O*-demethyltangeretin	*Vitex agnus-castus*	−61.5	−101.1
6,8-digalactosylapigenin	*Trigonella foenum-gracum*	no dock	no dock
diosmetin	*Turnera aphrodisiaca*	−87.8	−102.0
	*Turnera diffusa*		
epigallocatechin	*Ginkgo biloba*	−88.1	−80.5
eriodictyol	*Silybum marianum*	−89.1	−101.3
folerogenin	*Glycyrrhiza glabra*	−78.2	−71.4
8-galactopyranosyl-6-quinovopyranosylapigenin	*Trigonella foenum-graecum*	no dock	no dock
8-galactopyranosyl-6-xylopyranosylapigenin	*Trigonella foenum-graecum*	no dock	no dock
garbanzol	*Trifolium pratense*	−84.7	−83.6
ginkgetin	*Ginkgo biloba*	no dock	no dock
glabranin	*Glycyrrhiza glabra*	−90.3	−94.3
glabrol	*Glycyrrhiza glabra*	−96.1	−97.4
gonzalitosin	*Turnera aphrodisiaca*	−71.7	−106.3
	*Turnera diffusa*		
gossypetin	*Rhodiola rosea*	−98.6	−105.7
(2*R*,3*S*,4*S*)-3,3′,4,4′,5,5′,7-heptahydroxyflavan	*Ginkgo biloba*	−89.9	−82.8
herbacetin	*Rhodiola rosea*	−94.6	−102.1
3-hydroxyglabrol	*Glycyrrhiza glabra*	−68.9	−101.5
isocisilandrin	*Silybum marianum*	no dock	no dock
isoginkgetin	*Ginkgo biloba*	no dock	no dock
isolicoflavonol	*Glycyrrhiza glabra*	−97.0	−102.0
isoorientin	*Tribulus terrestris*	no dock	no dock
	*Trigonella foenum-graecum*		
	*Vitex agnus-castus*		
isorhamnetin	*Trifolium pratense*	−95.2	−103.4
isoschaftoside	*Glycyrrhiza glabra*	no dock	no dock
isosilandrin A	*Silybum marianum*	no dock	no dock
isosilandrin B	*Silybum marianum*	no dock	no dock
isosilybin A	*Silybum marianum*	no dock	no dock
isosilybin B	*Silybum marianum*	no dock	no dock
isosilybin C	*Silybum marianum*	no dock	no dock
isosilybin D	*Silybum marianum*	no dock	no dock
isosilychristin	*Silybum marianum*	no dock	−69.6
isoviolanthin	*Glycyrrhiza glabra*	−44.1	no dock
isovitexin	*Trigonella foenum-graecum*	no dock	−6.7
	*Vitex agnus-castus*		
isoxanthohumol	*Humulus lupulus*	−102.5	−99.6
kaempferol	*Ginkgo biloba*	−90.1	−98.8
	*Glycyrrhiza glabra*		
	*Silybum marianum*		
	*Tribulus terrestris*		
	*Trifolium pretense*		
	*Trigonella foenum-graecum*		
kumatakenin	*Glycyrrhiza glabra*	−88.3	−101.2
laricitrin	*Turnera aphrodisiaca*	−90.4	−107.3
	*Turnera diffusa*		
licoagrodin	*Glycyrrhiza glabra*	no dock	no dock
licoflavanone	*Glycyrrhiza glabra*	−95.2	−102.2
liquiritigenin	*Glycyrrhiza glabra*	−84.9	−97.1
luteolin	*Trigonella foenum-graecum*	−92.0	−103.5
	*Vitex agnus-castus*		
luteolin-8-propenoic acid	*Turnera aphrodisiaca*	−113.1	−123.1
	*Turnera diffusa*		
malvidin	*Trifolium pratense*	−86.0	−99.9
5′-methoxybilobetin	*Ginkgo biloba*	no dock	no dock
myricetin	*Trifolium pratense*	−90.7	−106.2
naringenin	*Glycyrrhiza glabra*	−86.3	−95.4
	*Silybum marianum*		
neocorymboside	*Trigonella foenum-graecum*	no dock	no dock
neosilyhermin A	*Silybum marianum*	−42.8	−44.5
neosilyhermin B	*Silybum marianum*	no dock	−67.6
norwogonin	*Glycyrrhiza glabra*	−83.7	−91.9
orientin	*Trigonella foenum-graecum*	−83.7	−79.2
	*Turnera aphrodisiaca*		
	*Turnera diffusa*		
	*Vitex agnus-castus*		
orientin-3*"*-ketone	*Turnera aphrodisiaca*	−83.8	−74.3
	*Turnera diffusa*		
pectolinarigenin	*Trifolium pratense*	−84.8	−99.7
peonidin	*Ginkgo biloba*	−94.0	−100.5
	*Trifolium pratense*		
pinocembrin	*Glycyrrhiza glabra*	−81.4	−88.1
	*Turnera aphrodisiaca*		
	*Turnera diffusa*		
6-prenyleriodictyol	*Glycyrrhiza glabra*	−82.1	−91.2
8-prenyleriodictyol	*Glycyrrhiza uralensis*	−102.9	−87.7
6-prenylnaringenin	*Glycyrrhiza glabra*	−62.8	−91.7
8-prenylnaringenin	*Humulus lupulus*	−99.2	−102.8
6-prenylpinocembrin	*Glycyrrhiza glabra*	−49.1	−89.1
quercetin	*Ginkgo biloba*	−94.5	−106.0
	*Glycyrrhiza glabra*		
	*Sambucus nigra*		
	*Silybum marianum*		
	*Tribulus terrestris*		
	*Trigonella foenum-graecum*		
rhodiolin	*Rhodiola rosea*	no dock	no dock
santin	*Vitex agnus-castus*	−31.3	−107.9
sciadopitysin	*Ginkgo biloba*	no dock	no dock
shinflavanone	*Glycyrrhiza glabra*	−63.1	−77.4
sigmoidin B	*Glycyrrhiza uralensis*	−35.5	−92.7
silandrin A	*Silybum marianum*	no dock	no dock
silandrin B	*Silybum marianum*	no dock	no dock
silyamandin	*Silybum marianum*	no dock	no dock
silybin A	*Silybum marianum*	no dock	no dock
silybin B	*Silybum marianum*	no dock	no dock
silychristin	*Silybum marianum*	no dock	no dock
silychristin B	*Silybum marianum*	−69.2	no dock
silydianin	*Silybum marianum*	no dock	−73.5
silyhermin	*Silybum marianum*	no dock	72.2
silymonin	*Silybum marianum*	no dock	−69.2
syringetin	*Turnera aphrodisiaca*	−80.5	−95.1
	*Turnera diffusa*		
taxifolin	*Silybum marianum*	−89.4	−104.6
3,4′,5,8-tetrahydroxyflavone	*Trifolium pratense*	−92.0	−103.1
tricetin	*Ginkgo biloba*	−86.2	−106.2
trigraecum	*Trigonella foenum-graecum*	−82.7	−94.4
vicenin 1	*Trigonella foenum-graecum*	no dock	no dock
vicenin 2	*Trigonella foenum-graecum*	no dock	no dock
vicenin 3	*Trigonella foenum-graecum*	no dock	no dock
vitexin	*Glycyrrhiza glabra*	−83.7	−88.5
	*Vitex agnus-castus*

**Table 7:** **MolDock molecular docking energies (kJ/mol) for isoflavonoids with human estrogen receptors α and β**

**Compound**	**Plant Source**	**ERα**	**ERβ**
7-acetoxy-2-methylisoflavone	*Glycyrrhiza glabra*	−78.4	−94.9
biochanin A	*Trifolium pratense*	−90.2	−98.6
calycosin	*Trifolium pratense*	−86.0	−103.3
daidzein	*Trifolium pratense*	−88.1	−95.0
formononetin	*Cimicifuga racemosa*	−83.5	−98.5
genistein	*Glycyrrhiza glabra*	−93.4	−98.9
	*Trifolium pratense*		
glabraisoflavanone A	*Glycyrrhiza glabra*	no dock	no dock
glabraisoflavanone B	*Glycyrrhiza glabra*	no dock	no dock
glabridin	*Glycyrrhiza glabra*	−15.8	−92.9
glabroisoflavanone A	*Glycyrrhiza glabra*	−35.5	−87.5
glabroisoflavanone B	*Glycyrrhiza glabra*	−29.1	−100.1
glabrone	*Glycyrrhiza glabra*	−39.0	−87.5
glyasperin B	*Glycyrrhiza glabra*	−81.7	−90.1
glyasperin K	*Glycyrrhiza glabra*	−35.4	−64.0
glyzaglabrin	*Glycyrrhiza glabra*	−99.1	−103.9
glyzarin	*Glycyrrhiza glabra*	−93.3	−94.2
7-hydroxy-2-methylisoflavone	*Glycyrrhiza glabra*	−79.0	−87.3
irilone	*Trifolium pratense*	−87.4	−102.8
isoderrone	*Glycyrrhiza glabra*	−27.5	−84.1
isoglabrone	*Glycyrrhiza glabra*	−34.1	−75.1
isomucronulatol	*Glycyrrhiza glabra*	−87.1	−104.4
kanzonol R	*Glycyrrhiza glabra*	−82.9	−101.7
kanzonol T	*Glycyrrhiza glabra*	no dock	no dock
kanzonol X	*Glycyrrhiza glabra*	−54.9	−51.2
licoagroside A (aglycone)	*Glycyrrhiza glabra*	−100.5	−107.7
licoricidin	*Glycyrrhiza glabra*	−41.5	no dock
lupiwighteone	*Glycyrrhiza glabra*	−96.1	−107.8
7-methoxy-2-methylisoflavone	*Glycyrrhiza glabra*	−79.2	−86.9
1-methoxyphaseollin	*Glycyrrhiza glabra*	−76.2	−110.5
phaseollinisoflavan	*Glycyrrhiza glabra*	−63.7	−89.4
pratensein	*Trifolium pratense*	−93.7	−106.4
8-prenylphaseollinisoflavan	*Glycyrrhiza glabra*	no dock	−43.2
prunetin	*Glycyrrhiza glabra*	−92.8	−98.9
pseudobaptigenin	*Trifolium pratense*	−94.6	−104.1
shinpterocarpin	*Glycyrrhiza glabra*	−65.3	−95.1
2′,4′,5,7-tetrahydroxy-3′,8-diprenylisoflavanone	*Glycyrrhiza glabra*	−37.0	−34.8
tetrapterol G	*Glycyrrhiza glabra*	−49.9	−100.8
3′,5,7-trihydroxy-5′-methoxyisoflavone	*Trigonella foenum-graecum*	−97.4	−107.9
wighteone	*Glycyrrhiza glabra*	−75.8	−101.9
vitexcarpan	*Vitex agnus-castus*	−70.4	−99.5

**Table 8:** **MolDock molecular docking energies (kJ/mol) for lignans with human estrogen receptors α and β**

Compound	Plant Source	ERα	ERβ
actaealactone	*Cimicifuga racemosa*	−102.8	−112.3
(−)-arctigenin	*Arctium lappa*	−109.9	−116.2
(*Z*)-dehydrodiconiferyl alcohol	*Silybum marianum*	−97.9	−110.6
dihydrodehydrodiconiferyl alcohol (9-acetate)	*Sambucus nigra*	−102.7	−97.5
7′-hydroxymatairesinol	*Podocarpus spicatus*	−112.3	−117.3
isolariciresinol	*Picea excelsa*	−76.2	−84.6
(+)-lariciresinol	*Rhodiola rosea*	−104.2	−113.7
licoagrocarpin	*Glycyrrhiza glabra*	−90.4	−85.4
nordihydroguaiaretic acid	*Guaiacum officinale*	−102.1	−106.4
(−)-nortrachelogenin	*Pinus palustris*	−112.0	−125.4
pinoresinol	*Picea excelsa*	−106.4	−117.7
secoisolariciresinol	*Picea abies*	−109.1	−114.2
sesamin	*Ginkgo biloba*	−99.1	−121.8

**Table 9:** **MolDock molecular docking energies (kJ/mol) for phenanthrenoids with human estrogen receptors α and β**

**Compound**	**Plant Source**	**ERα**	**ERβ**
batatasin I	*Dioscorea* spp.	−84.7	−97.1
denthyrsinin	*Dioscorea* spp.	−83.5	−96.7
9,10-dihydro-2,7-dihydroxy-1,3,5-trimethoxyphenanthrene	*Dioscorea* spp.	−80.8	−95.7
9,10-dihydro-5,7-dimethoxy-3,4-phenanthrenediol	*Dioscorea* spp.	−81.0	−90.1
9,10-dihydro-2,3,5,7-phenanthrenetetrol	*Dioscorea* spp.	−78.0	−83.2
9,10-dihydro-4,6,7-trimethoxy-2-phenanthrenol	*Dioscorea* spp.	−88.5	−99.6
9,10-dihydro-5,6,8-trimethoxy-3,4-phenanthrenediol	*Dioscorea* spp.	−72.9	−87.5
6,7-dihydroxy-2-methoxy-1,4-phenanthraquinone	*Dioscorea* spp.	−85.9	−94.9
3,5-dimethoxy-2,7-phenanthrenediol	*Dioscorea* spp.	−84.6	−93.5
5,7-dimethoxy-2,3-phenanthrenediol	*Dioscorea* spp.	−88.5	−93.6
diobulbinone	*Dioscorea* spp.	no dock	no dock
dioscoreanone	*Dioscorea* spp.	−88.6	−96.1
hircinol	*Dioscorea* spp.	−68.9	−76.3
6-methoxycoelonin	*Dioscorea* spp.	−83.5	−93.9
2,4,5,6-phenanthrenetetrol	*Dioscorea* spp.	−74.7	−82.9
3,4,6-phenanthrenetriol	*Dioscorea* spp.	−74.3	−79.9
prazerol	*Dioscorea* spp.	−84.6	−96.5

**Table 10:** **MolDock molecular docking energies (kJ/mol) for miscellaneous phenolic ligands with human estrogen receptors α and β**

**Compound**	**Plant Source**	**ERα**	**ERβ**
agnucastoside C (aglycone)	*Vitex agnus-castus*	−106.9	−130.0
agnuside (aglycone)	*Vitex agnus-castus*	−103.1	−110.3
angeliferulate	*Angelica sinensis*	−110.7	−121.5
1,3-bis(2,4-dihydroxyphenyl)propane	*Dioscorea* spp.	−101.7	−101.1
1,3-bis(2-hydroxy-4-methoxyphenyl)propane	*Dioscorea* spp.	−97.1	−94.9
burkinabin A	*Echinacea* spp.	−97.6	−100.6
burkinabin B	*Echinacea* spp.	−101.2	−85.1
caffeic acid	*Echinacea* spp.	−72.1	−74.5
caffeoyl-*p*-coumaroyltartaric acid	*Echinacea* spp.	−98.3	−129.8
caffeoylferuloyltartaric acid	*Echinacea* spp.	−76.1	−96.2
*trans*-caffeoylglycolic acid	*Cimicifuga racemosa*	−87.3	−96.7
caftaric acid	*Echinacea* spp.	−105.7	−112.1
chicoric acid	*Echinacea* spp.	−99.1	−116.1
cimicifugic acid A	*Cimicifuga racemosa*	−102.9	−124.1
cimicifugic acid B	*Cimicifuga racemosa*	−114.4	−120.5
cimicifugic acid F	*Cimicifuga racemosa*	−126.2	−125.2
cimicifugic acid G	*Cimicifuga racemosa*	−101.4	−113.4
cimiciphenol	*Cimicifuga racemosa*	−113.2	−119.4
cimiciphenone	*Cimicifuga racemosa*	−109.0	−120.8
cimifugin	*Cimicifuga racemosa*	−93.6	−97.1
cimiracemate A	*Cimicifuga racemosa*	−113.9	−120.9
cimiracemate B	*Cimicifuga racemosa*	−114.4	−127.3
cimiracemate C	*Cimicifuga racemosa*	−110.5	−115.8
cimiracemate D	*Cimicifuga racemosa*	−104.2	−128.5
*p*-coumaric acid	*Trigonella foenum-graecum*	−65.8	−69.0
diferuloyltartaric acid	*Echinacea* spp.	−102.6	−88.7
6,7-dihydroxy-1,1-dimethylisochroman	*Dioscorea* spp.	−68.4	−67.5
2-(3,7-dimethyl-2,6-octadienyl)-4-hydroxy-6-methoxyacetophenone	*Dioscorea* spp.	−99.6	−104.8
ferulic acid	*Echinacea* spp.	−70.7	−78.2
fukiic acid	*Cimicifuga racemosa*	−83.4	−88.1
fukinolic acid	*Cimicifuga racemosa*	−113.6	−127.3
irbic acid	*Centella asiatica*	no dock	−17.6
isoferulic acid	*Cimicifuga racemosa*	−68.6	−73.7
licoagroaurone	*Glycyrrhiza glabra*	−109.5	−117.9
licoagrone	*Glycyrrhiza glabra*	no dock	no dock
paeonol	*Dioscorea* spp.	−59.4	−62.5
(*S*)-phaselic acid	*Trifolium pratense*	−99.7	−109.6
trichocarpinine	*Echinacea* spp.	−84.4	−92.4

**Table 11:** **MolDock molecular docking energies (kJ/mol) for sesquiterpenoids with human estrogen receptors α and β**

**Compound**	**Plant Source**	**ERα**	**ERβ**
bilobanol	*Ginkgo biloba*	−84.8	−89.2
bisabolangelone	*Angelica sinensis*	−80.2	−90.4
cinnamoyldihydroxynardol	*Echinacea* spp.	−99.5	−98.5
cinnamoylechinadiol	*Echinacea* spp.	−83.6	−120.8
cinnamoylechinaxanthol	*Echinacea* spp.	−87.6	−94.9
cinnamoylepoxyechinadiol	*Echinacea* spp.	−86.4	−107.9

**Table 12:** **MolDock molecular docking energies (kJ/mol) for steroids with human estrogen receptors α and β**

**Compound**	**Plant Source**	**ERα**	**ERβ**
campesterol	*Centella asiatica*	−71.1	−108.8
	*Ptychopetalum olacoides*		
	*P. uncinatum*		
	*Sambucus nigra*		
	*Tribulus terrestris*		
	*Trifolium pratense*		
chiapagenin	*Dioscorea* spp.	−61.5	no dock
chlorogenin	*Tribulus terrestris*	−5.7	no dock
cholest-5-ene-3,12,16,22-tetrol	*Dioscorea* spp.	−49.5	−82.0
correllogenin	*Dioscorea* spp.	−64.3	no dock
cryptogenin	*Dioscorea* spp.	−51.3	−74.8
2,3-dihydroxypregn-16-en-20-one	*Tribulus terrestris*	−105.3	−116.6
2,3-dihydroxyspirost-4-en-12-one	*Tribulus terrestris*	no dock	no dock
3,16-dihydroxypregn-5-en-20-one	*Dioscorea* spp.	−91.9	−116.6
3,16-dihydroxypregnane-12,20-dione	*Tribulus terrestris*	−93.7	−95.6
3,21-dihydroxypregna-5,16-dien-20-one	*Dioscorea* spp.	−103.1	−121.4
diosbulbisin A	*Dioscorea* spp.	no dock	no dock
diosbulbisin B	*Dioscorea* spp.	no dock	no dock
diosbulbisin C	*Dioscorea* spp.	no dock	no dock
diosbulbisin D	*Dioscorea* spp.	−59.9	no dock
diosgenin	*Dioscorea* spp.	−58.9	no dock
	*Tribulus terrestris*		
	*Trigonella foenum-graecum*		
diosgenin acetate	*Dioscorea* spp.	no dock	no dock
diotigenin	*Dioscorea* spp.	no dock	no dock
doristerol	*Dioscorea* spp.	−62.1	−80.3
episarsasapogenin	*Dioscorea* spp.	no dock	no dock
epismilagenin	*Dioscorea* spp.	−53.4	no dock
ergost-5-ene-3,26-diol	*Dioscorea* spp.	−71.3	−108.6
ergost-8(14)-en-3-ol	*Dioscorea* spp.	−69.5	−98.5
furost-20(22)-ene-2,3,26-triol	*Tribulus terrestris*	−37.8	−44.1
furost-20(22)-ene-3,26-diol	*Tribulus terrestris*	−10.1	−45.5
furost-5-ene-3,16,26-triol	*Tribulus terrestris*	−49.7	−45.8
furost-5-ene-3,22,26,27-tetrol	*Dioscorea* spp.	−35.1	−4.6
furost-5-ene-3,22,26-triol	*Dioscorea* spp.	−48.2	−24.1
	*Tribulus terrestris*		
furosta-5,20(22)-diene-3,26-diol	*Dioscorea* spp.	−30.4	−36.0
furostane-1,2,3,22,26-pentol	*Dioscorea* spp.	−40.7	−51.4
gentrogenin	*Dioscorea* spp.	−49.8	no dock
gitogenin	*Tribulus terrestris*	no dock	no dock
	*Trigonella foenum-graecum*		
globosterol	*Ginkgo biloba*	−31.4	−11.3
hecogenin	*Tribulus terrestris*	no dock	no dock
26-hydroxyfurosta-4,20(22)-diene-3,12-dione	*Tribulus terrestris*	−20.2	−32.5
24-hydroxyspirost-4-ene-3,12-dione	*Tribulus terrestris*	no dock	no dock
19-hydroxyyonogenin	*Dioscorea* spp.	no dock	no dock
igagenin	*Dioscorea* spp.	−41.8	no dock
isochiapagenin	*Dioscorea* spp.	−49.1	no dock
isodiotigenin	*Dioscorea* spp.	−54.5	no dock
isonarthogenin	*Dioscorea* spp.	−61.3	no dock
kogagenin	*Dioscorea* spp.	−56.5	no dock
marianine	*Silybum marianum*	−5.9	−57.3
24-methylenelanost-8-ene-3,25,28-triol	*Silybum marianum*	−29.3	−38.3
neogitogenin	*Tribulus terrestris*	−55.1	no dock
neohecogenin	*Tribulus terrestris*	−39.7	no dock
neokammogenin	*Dioscorea* spp.	no dock	no dock
neotigogenin	*Tribulus terrestris*	−51.9	no dock
	*Trigonella foenum-graecum*		
neoyonogenin	*Dioscorea* spp.	no dock	no dock
pentandroside F (aglycone)	*Tribulus terrestris*	−37.5	−41.4
	*Trigonella foenum-graecum*		
prazerigenin A	*Dioscorea* spp.	−56.1	no dock
prazerigenin B	*Dioscorea* spp.	−53.2	no dock
prazerigenin C	*Dioscorea* spp.	−60.7	no dock
pregnadienolone	*Dioscorea* spp.	−102.7	−115.4
protoyonogenin (aglycone)	*Dioscorea* spp.	−28.9	−17.8
ruscogenin	*Tribulus terrestris*	−6.9	no dock
sarsasapogenone	*Dioscorea* spp.	−51.4	no dock
β-sitosterol	*Tribulus terrestris*	−65.0	−102.8
smilagenone	*Dioscorea* spp.	no dock	no dock
25*R*-spirosta-3,5-diene	*Trigonella foenum-graecum*	−54.8	no dock
spirost-4-ene-3,12-dione	*Tribulus terrestris*	−67.9	no dock
spirost-4-ene-3,6,12-trione	*Tribulus terrestris*	no dock	no dock
spirosta-3,5-dien-12-one	*Tribulus terrestris*	−50.5	no dock
spirostane-3,23,24-triol	*Tribulus terrestris*	no dock	no dock
spirostane-3,6,12-trione	*Tribulus terrestris*	no dock	no dock
steroid G4	*Dioscorea* spp.	no dock	no dock
stigmast-8(14)-en-3-ol	*Dioscorea* spp.	−45.3	−75.3
terrestrosin K (aglycone)	*Tribulus terrestris*	no dock	−60.5
1,2,3,16-tetrahydroxypregnan-20-one	*Dioscorea* spp.	−88.7	−48.9
tigogenin	*Tribulus terrestris*	no dock	no dock
	*Trigonella foenum-graecum*		
tokorogenin	*Dioscorea* spp.	−50.3	no dock
tribufuroside C (aglycone)	*Tribulus terrestris*	no dock	−65.4
tribufuroside D (aglycone)	*Tribulus terrestris*	no dock	−13.1
tribufuroside I (aglycone)	*Tribulus terrestris*	−2.9	−35.0
tribufuroside J (aglycone)	*Tribulus terrestris*	no dock	−34.3
trigoneoside (aglycone)	*Trigonella foenum-graecum*	−53.8	−55.6
2,3,4-trihydroxypregn-16-en-20-one	*Dioscorea* spp.	−82.7	−71.9
yamogenin	*Dioscorea* spp.	−59.4	no dock
	*Trigonella foenum-graecum*		
yonogenin	*Dioscorea* spp.	no dock	no dock

**Table 13:** **MolDock molecular docking energies (kJ/mol) for stilbenoids with human estrogen receptors α and β**

**Compound**	**Plant Source**	**ERα**	**ERβ**
3-acetoxy-4′,5-dihydroxy-3′-prenyldihydrostilbene	*Glycyrrhiza glabra*	−119.0	−118.7
batatasin II	*Dioscorea* spp.	−84.6	−94.7
batatasin III	*Dioscorea* spp.	−83.3	−92.6
batatasin IV	*Dioscorea* spp.	−80.7	−93.4
batatasin V	*Dioscorea* spp.	−88.6	−98.4
demethylbatatasin IV	*Dioscorea* spp.	−81.1	−92.3
dihydropinosylvin	*Dioscorea* spp.	−77.9	−86.2
dihydropinosylvin methyl ether	*Dioscorea* spp.	−74.1	−89.3
dihydroresveratrol	*Dioscorea* spp.	−83.0	−94.4
2,4′-dihydroxy-3′,5′-dimethoxybibenzyl	*Dioscorea* spp.	−87.4	−100.3
gancaonin R	*Glycyrrhiza uralensis*	−102.1	−107.0
licoagrodione	*Glycyrrhiza glabra*	−98.2	−116.9
piceatannol	*Picea abies*	−85.9	−100.3
3,3′,4,5′-tetrahydroxy-4′,5-diprenylbibenzyl	*Glycyrrhiza glabra*	−108.9	−117.1
2,2′,5,5′-tetrahydroxy-3-methoxybibenzyl	*Dioscorea* spp.	−87.4	−99.0
3,3′,4,5′-tetrahydroxy-5-prenylbibenzyl	*Glycyrrhiza glabra*	−111.4	−115.0
3,3′,5′-trihydroxy-4-methoxybibenzyl	*Glycyrrhiza glabra*	−88.0	−97.7
3,4′,5-trihydroxy-3′,4-diprenylbibenzyl	*Glycyrrhiza glabra*	−107.4	−111.0
3,3′,5′-trihydroxy-4-methoxy-5-prenylbibenzyl	*Glycyrrhiza glabra*	−111.4	−113.6
3,4′,5-trihydroxy-3′-prenyldihydrostilbene	*Glycyrrhiza glabra*	−106.3	−112.0
tristin	*Dioscorea* spp.	−91.1	−98.7
uralstilbene	*Glycyrrhiza glabra*	−101.7	−122.1

**Table 14:** **MolDock molecular docking energies (kJ/mol) for triterpenoids with human estrogen receptors α and β**

**Compound**	**Plant Source**	**ERα**	**ERβ**
actaeaepoxide	*Cimicifuga racemosa*	no dock	no dock
acteol	*Cimicifuga racemosa*	−18.4	no dock
acteol-12-acetate	*Cimicifuga racemosa*	no dock	no dock
acteol-26-ketone	*Cimicifuga racemosa*	−42.3	no dock
12β-acetoxycimigenol	*Cimicifuga racemosa*	no dock	no dock
25-acetoxy-12β-hydroxycimigenol	*Cimicifuga racemosa*	no dock	no dock
24-acetoxyisodahurinol	*Cimicifuga racemosa*	no dock	no dock
23-acetoxyshengmanol	*Cimicifuga racemosa*	no dock	no dock
3′-acetylcimicifugoside (aglycone)	*Cimicifuga racemosa*	−30.4	−25.5
α-amyrin	*Sambucus nigra*	no dock	−5.6
β-amyrin	*Glycyrrhiza glabra*	no dock	no dock
α-amyrone	*Sambucus nigra*	no dock	no dock
25-anhydrocimigenol-12β-acetoxy	*Cimicifuga racemosa*	no dock	no dock
asiatic acid	*Centella asiatica*	no dock	no dock
asiaticoside G (aglycone)	*Centella asiatica*	no dock	no dock
betulafolienetriol	*Centella asiatica*	−73.1	−67.8
betulin	*Sambucus nigra*	no dock	−26.3
betulinic acid	*Glycyrrhiza glabra*	no dock	−43.9
caulophyllogenin	*Cimicifuga racemosa*	no dock	no dock
centellasapogenol A	*Centella asiatica*	no dock	no dock
centelloside A (aglycone)	*Centella asiatica*	−59.8	−75.5
cimicidol-3-one	*Cimicifuga racemosa*	−47.0	−65.6
cimigenol	*Cimicifuga racemosa*	no dock	no dock
cimipodocarpaside (aglycone)	*Cimicifuga racemosa*	−28.8	−4.4
cimiracemoside F (aglycone)	*Cimicifuga racemosa*	no dock	no dock
cimiracemoside H (aglycone)	*Cimicifuga racemosa*	no dock	no dock
cimiracemoside I (aglycone)	*Cimicifuga racemosa*	−66.2	no dock
corosolic acid	*Centella asiatica*	no dock	no dock
9(11)-dehydroglycyrrhetic acid	*Glycyrrhiza glabra*	no dock	no dock
26-deoxyacteol	*Cimicifuga racemosa*	−39.7	no dock
11-deoxoglycyrrhetic acid	*Glycyrrhiza glabra*	no dock	no dock
desoxoglabrolide	*Glycyrrhiza glabra*	no dock	no dock
12β,21-dihydroxycimigenol	*Cimicifuga racemosa*	no dock	no dock
2,3-dihydroxy-5-(hydroxymethyl)-24-norolean-12-en-28-oic acid	*Centella asiatica*	no dock	no dock
3,24-dihydroxy-11,13(18)-oleanadien-30-oic acid methyl ester	*Glycyrrhiza glabra*	no dock	no dock
3,24-dihydroxy-9(11),12-oleanadien-30-oic acid	*Glycyrrhiza glabra*	no dock	no dock
23-*epi*-26-deoxyacteol	*Cimicifuga racemosa*	−53.0	no dock
glabric acid	*Glycyrrhiza glabra*	no dock	no dock
glabrolide	*Glycyrrhiza glabra*	no dock	no dock
glycyrrhetic acid	*Glycyrrhiza glabra*	no dock	no dock
18α-glycyrrhetic acid	*Glycyrrhiza glabra*	no dock	no dock
glycyrrhetol	*Glycyrrhiza glabra*	no dock	no dock
21-hydroxycimigenol	*Cimicifuga racemosa*	no dock	no dock
18α-hydroxyglycyrrhetic acid	*Glycyrrhiza glabra*	no dock	no dock
24-hydroxyglycyrrhetic acid	*Glycyrrhiza glabra*	no dock	no dock
28-hydroxyglycyrrhetic acid	*Glycyrrhiza glabra*	no dock	no dock
21-hydroxyisoglabrolide	*Glycyrrhiza glabra*	no dock	no dock
24-hydroxyliquiritic acid	*Glycyrrhiza glabra*	no dock	no dock
6β-hydroxymaslinic acid	*Centella asiatica*	no dock	no dock
isoglabrolide	*Glycyrrhiza glabra*	no dock	no dock
isothankunic acid	*Centella asiatica*	no dock	no dock
lanosta-5,24-dien-3-ol	*Glycyrrhiza glabra*	−55.6	−52.3
liquiridiolic acid	*Glycyrrhiza glabra*	no dock	no dock
liquiritic acid	*Glycyrrhiza glabra*	no dock	no dock
liquoric acid	*Glycyrrhiza glabra*	no dock	no dock
lupeol	*Ptychopetalum olacoides*	no dock	−40.5
	*P. uncinatum*		
	*Sambucus nigra*		
madasiatic acid	*Centella asiatica*	no dock	no dock
madecassic acid	*Centella asiatica*	no dock	no dock
neocimicigenol	*Cimicifuga racemosa*	no dock	no dock
oleanolic acid	*Sambucus nigra*	no dock	no dock
quasipanaxadiol	*Centella asiatica*	no dock	−35.5
shengmanol	*Cimicifuga racemosa*	no dock	−3.0
silymin A	*Sambucus nigra*	no dock	no dock
silymin B	*Sambucus nigra*	no dock	no dock
terminolic acid	*Centella asiatica*	no dock	no dock
2,3,20,23-tetrahydroxy-28-ursanoic acid	*Centella asiatica*	no dock	no dock
2,3,23-trihydroxy-20-ursen-28-oic acid	*Centella asiatica*	no dock	no dock
3,6,23-trihydroxy-12-ursen-28-oic acid	*Centella asiatica*	no dock	no dock
uncargenin C	*Centella asiatica*	no dock	no dock
ursolic acid	*Sambucus nigra*	no dock	no dock
zemoside A (aglycone)	*Centella asiatica*	no dock	no dock

**Table 15:** **MolDock molecular docking energies (kJ/mol) for miscellaneous phytochemicals with human estrogen receptors α and β**

**Compound**	**Plant Source**	**ERα**	**ERβ**
10-angeloylbutylphthalide	*Angelica sinensis*	−96.9	−107.1
ansaspirolide	*Angelica sinensis*	−98.5	−91.5
asiaticin	*Centella asiatica*	−96.7	−109.0
3a,7′a:7a,3′a-diligustilide	*Angelica sinensis*	−94.5	−69.6
3a,8′:6,3′-diligustilide	*Angelica sinensis*	−91.3	−72.6
3a,8′:6,3′-diligustilidetriepimer	*Angelica sinensis*	−98.0	−78.1
dioscorealide A	*Dioscorea* spp.	−92.1	−97.1
dioscorealide B	*Dioscorea* spp.	−86.2	−98.7
diospongin A	*Dioscorea* spp.	−95.1	−104.8
diospongin B	*Dioscorea* spp.	−97.1	−103.9
diospongin C	*Dioscorea* spp.	−96.1	−107.9
gelispirolide	*Angelica sinensis*	−64.7	−84.8
homosenkyunolide H	*Angelica sinensis*	−83.8	−88.7
homosenkyunolide I	*Angelica sinensis*	−84.3	−92.2
homosilphiperfoloic acid	*Centella asiatica*	−83.0	−77.3
levistolide A	*Angelica sinensis*	−86.5	−86.4
neodiligustilide	*Angelica sinensis*	−78.3	−6.6
orobanchyl acetate	*Trifolium pratense*	−111.3	−122.8
riligustilide	*Angelica sinensis*	−71.2	−81.6
senkyunolide O	*Angelica sinensis*	−84.8	−91.3
sinaspirolide	*Angelica sinensis*	−86.8	−66.2
3,3a,7a,8-tetrahydro-3,6′:7a,7′-diligustilid-8-one	*Angelica sinensis*	−80.8	−99.2
estradiol	Positive control	−92.0	−100.0
zearalenone	Positive control	−104.1	−104.9

## Results

### Alkaloids

**Figure 44:**
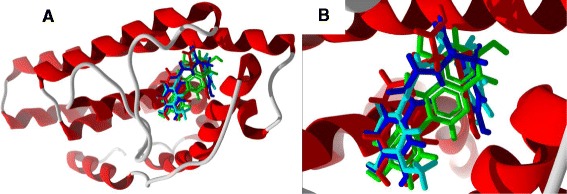
**Lowest-energy docked poses of alkaloids [** ***N-trans*** **-feruloyltyramine (aqua),** ***cis-*** **clovamide (red), and** ***trans-*** **clovamide (blue) along with the co-crystallized ligand, genistein (green)] with ERα (PDB 1X7R). A:** Docked poses showing the entire ribbon structure of the protein. **B:** Close-up of the docked poses.

**Figure 45:**
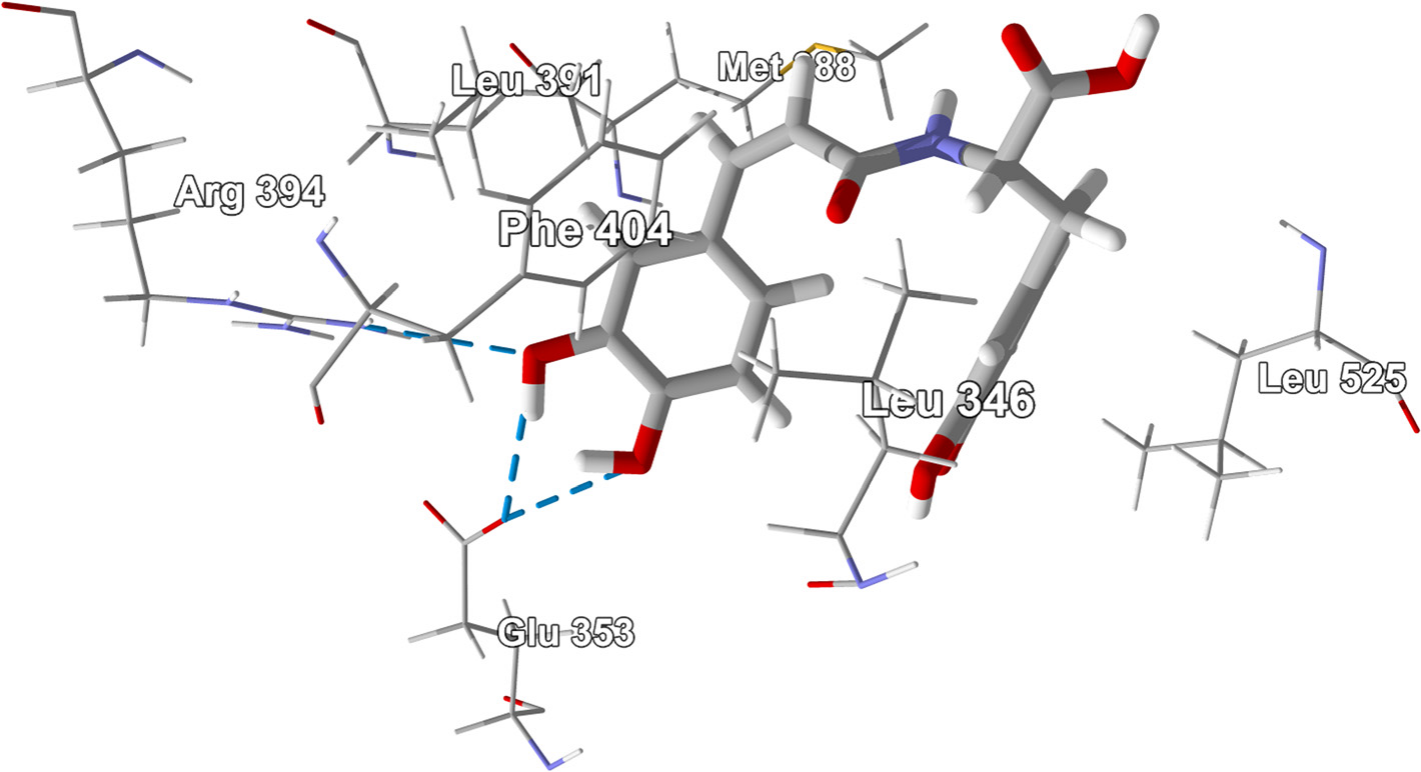
**Lowest-energy docked pose of** ***cis-*** **clovamide with ERα (PDB 1X7R) showing the principle amino acid contacts in the binding site.** Hydrogen bonds are indicated by blue dashed lines.

The alkaloid ligands examined in this study are shown in Figure 2. The molecular docking results for the alkaloids are summarized in Table 2. Of the alkaloids examined in this study, *cis-* and *trans-*clovamide, with docking energies of −119.8 and −113.6 kJ/mol, respectively, and *N-trans-*feruloyltyramine (*E*_dock_ = −103.1 kJ/mol) were found to dock well with ERα. Their docking energies were more exothermic than those of estradiol, −92.0 kJ/mol and the corresponding co-crystallized ligand genistein, −93.4 kJ/mol, and the clovamides were more exothermic than zearalenone (*E*_dock_ = −104.1 kJ/mol). The co-crystallized ligand, genistein, and the clovamide and feruloylyramine ligands have similar positions in the binding site (Figure 44). Phe 404, Leu 525, Leu 346, Leu 387, and Leu 391 form a hydrophobic pocket around the docked alkaloids. Phe 404 exhibited edge-to-face π–π interactions between the phenyl substituent of Phe with the caffeic or ferulic substituents of the alkaloids and with the hydroxyphenyl substituent of genistein. Notable hydrogen bonds in the lowest-energy docked pose of *cis*-clovamide were the 3-OH and 4-OH of the *cis*-caffeic moiety with the carboxylate residue of Glu 353 and the 3-OH group with the guanidine residue of Arg 394 (Figure 45). The docked *trans-*clovamide had hydrogen bonds between the 4-OH of the caffeate with the guanidine of Arg 394 and the carbonyl group of Leu 387 and the 3-OH group with the carboxylate of Glu 353. Hydrogen bonds were formed between the 4-OH group on the ferulyl substituent of *N-trans-*feruloyltyramine and carbonyl group of Leu 387, the guanidine group of Arg 394, and the carboxylate of Glu 353.

**Figure 46:**
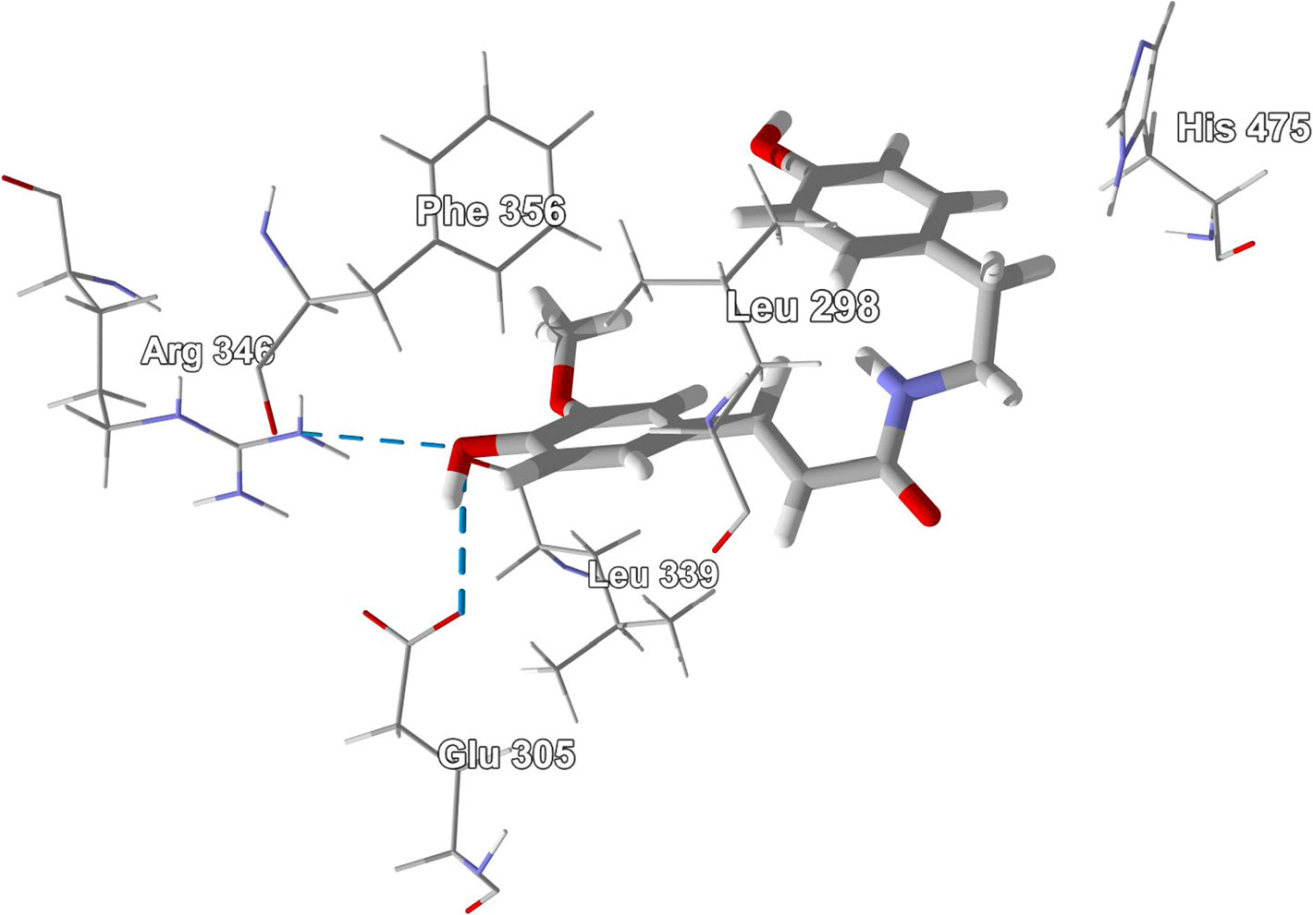
**Lowest-energy docked pose of** ***N-trans-*** **feruloyltyramine with ERβ (PDB 1X7B) showing the principle amino acid contacts in the binding site.** Hydrogen bonds are indicated by blue dashed lines.

Similarly, *cis-*clovamide, *trans-*clovamide, and *N-trans-*feruloyltyramine were the alkaloids that docked well with ERβ. Their docking energies (−124.9, −122.0, and −113.8 kJ/mol, respectively) were more exothermic than those of estradiol, −100.0 kJ/mol, zearalenone, −104.9 kJ/mol, and the corresponding co-crystallized ligand 2-(3-fluoro-4-hydroxyphenyl)-7-vinyl-1,3-benzoxazol-5-ol, −107.9 kJ/mol. The alkaloid and the co-crystallized ligand occupied similar positions in the binding site, a hydrophobic pocket formed by Leu 298, Phe 356, Leu 339, and His 475. Phe 356 exhibited edge-to-face π–π interactions with the caffeic or ferulic substituents of the docked alkaloid ligands as well as with the hydroxyphenyl substituent of the co-crystallized ligand. There were two notable hydrogen bonds formed between the 4-OH group on the ferulyl substituent of *N-trans-*feruloyltyramine and the guanidine group of Arg 346, and the carboxylate of Glu 305 (Figure 46). These same two residues formed hydrogen bonds with the 4-hydroxyphenyl group of the co-crystallized ligand. The caffeoyl group of *cis*-clovamide formed hydrogen bonds with Glu 305 and Leu 298. *trans-*Clovamide, however, formed hydrogen bonds with Leu 339, Arg 346, and Glu 305.

### Chalcones

The structures of the chalcones are shown in Figure 3, while the docking energies are summarized in Table 3. Xanthohumol was the strongest docking chalcone with ERα. Its docking energy, −116.8 kJ/mol, is more exothermic than those of estradiol, −92.0 kJ/mol, zearalenone, −104.1 kJ/mol, and the corresponding co-crystallized ligand genistein, −93.4 kJ/mol. The 4-OH group of xanthohumol forms three hydrogen-bonds with the protein (the carboxylate of Glu 353, the guanidine of Arg 394, and the carbonyl oxygen of Phe 404). The 4′-OH group of xanthohumol forms hydrogen-bonds with the imidazole N-H of His 524 and the carbonyl oxygen of Gly 521. Kanzonol Y (*E*_dock_ = −111.2 kJ/mol) and licochalcone B (*E*_dock_ = −107.8 kJ/mol) were the only other chalcone ligands to dock well with ERα.

**Figure 47:**
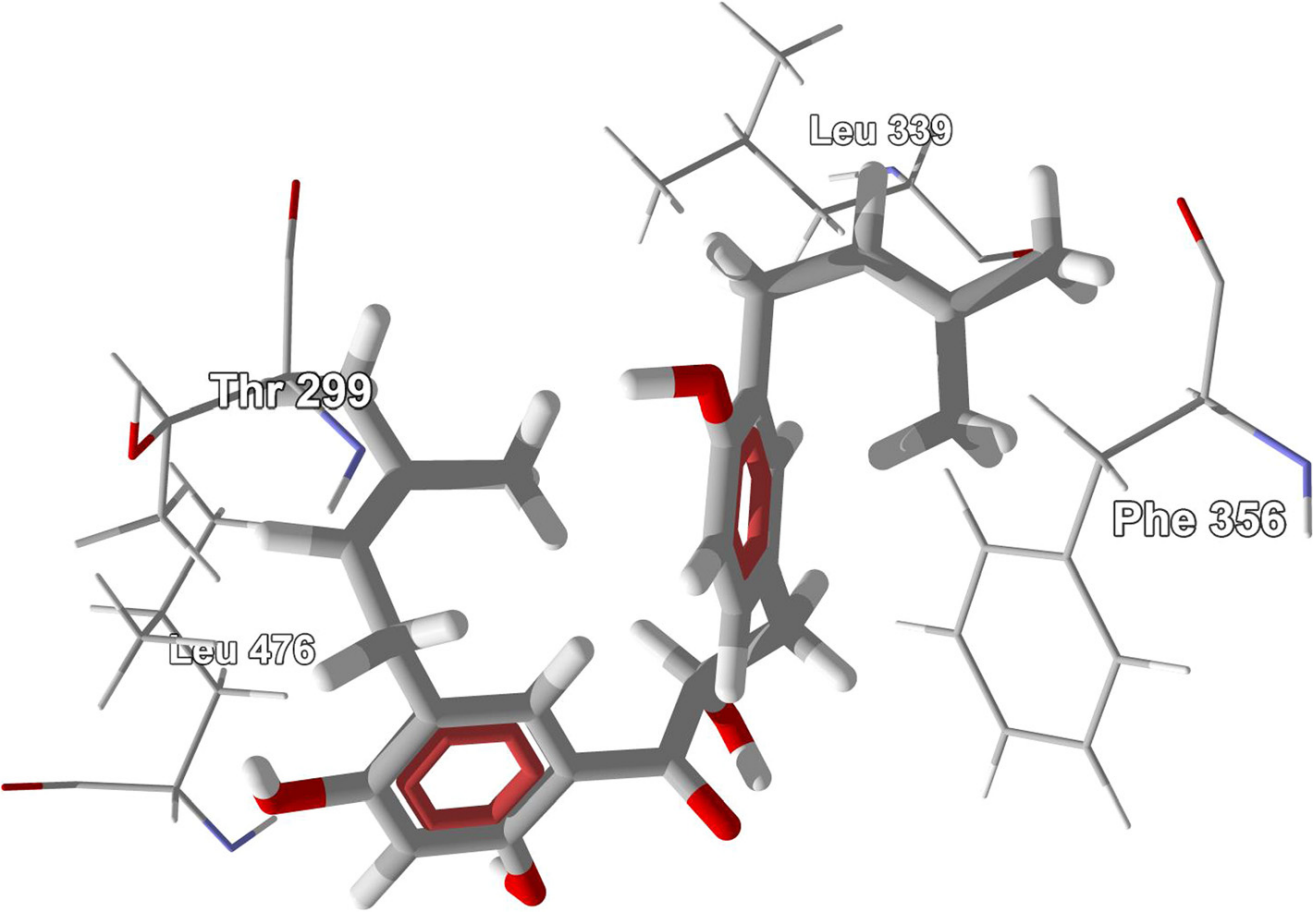
**Lowest-energy docked pose of the prenylated chalcone kanzonol Y with ERβ (PDB 1X7J) showing the hydrophobic amino acid contacts with the isoprenyl groups of the ligand.**

Of the chalcone ligands examined, kanzonol Y (*E*_dock_ = −122.4 kJ/mol), xanthohumol (*E*_dock_ = −116.8 kJ/mol), and licoagrochalcone A (*E*_dock_ = −115.5 kJ/mol), docked best with ERβ. Their docking energies were decidedly more exothermic than those of estradiol, zearalenone, and the corresponding co-crystallized ligand 2-(5-hydroxy-naphthalen-1-yl)-1,3-benzooxazol-6-ol (*E*_dock_ = −109.2 kJ/mol). Apparently, the hydrophobic prenyl groups allow for stronger docking. Thus, kanzonol Y docked to ERβ much better than the non-prenylated α,2′,4,4′-tetrahydroxydihydrochalcone (*E*_dock_ = −105.0 kJ/mol). In the lowest-energy docked pose of kanzonol Y, the 3-prenyl group is sandwiched between the hydrophobic residues of Phe 356 and Leu 339, while the 5′-prenyl group is sandwiched between Leu 476 and Thr 299 (Figure 47). It has been shown that prenylation of flavonoids and related compounds does alter the estrogenic activity and often results in antiestrogenic activity (Kretzschmar et al. 2010; Simons et al. 2012).

### Coumarins

The MolDock docking energies of the coumarins are summarized in Table 4, and the structures of the coumarins are shown in Figures 4 and 5. Glabrene and pratenol B were the strongest docking coumarins with ERα (*E*_dock_ = −104.8 kJ/mol), more exothermic than either estradiol (−92.0 kJ/mol) or genistein (−93.4 kJ/mol), and comparable to zearalenone (−104.1 kJ/mol). The 7-hydroxychromene moieties of both glabrene and pratenol B are held in a hydrophobic pocket formed by Leu 387, Phe 404, Met 388, and Leu 391. Furthermore, there are edge-to-face π–π interactions between Phe 404 and the chromene benzene rings of the ligands, as well as hydrogen bonds between the 7-OH group of the chromene and the guanidine group of Arg 394 and the carboxylate of Glu 356, analogous to the co-crystallized ligand genistein. Additionally, one of the carboxylates of pratenol B forms a hydrogen bond with imidazole substituent NH group of His 475.

In addition to glabrene (*E*_dock_ = −114.9 kJ/mol) and pratenol B (*E*_dock_ = −112.0 kJ/mol), mirificoumestan docked strongly with ERβ with a docking energy of −113.0 kJ/mol. These compounds docked more exothermically than estradiol, zearalenone, or the co-crystallized ligand [5-hydroxy-2-(4-hydroxyphenyl)-1-benzofuran-7-yl]acetonitrile (*E*_dock_ = −107.7 kJ/mol). Glabrene docked into the hydrophobic pocket formed by Leu 298, Leu 339, and Phe 356. Phe 356 exhibited edge-to-face π–π interactions with the hydroxychromene substituent of glabrene. There was a hydrogen bond between the imidazole substituent NH group of His 475 and the 5′-OH group of glabrene, and three hydrogen bonding interactions were seen between the 7-OH group of glabrene and the carbonyl group of Leu 339, the guanidine moiety of Arg 346, and the carboxylate of Glu 305.

### Diterpenoids

The structures for the diterpenoid ligands examined in this work are shown in Figures 6, 7, 8, and 9, and the docking energies are listed in Table 5. The strongest docking diterpenoids with ERα were diosbulbin F, diosbulbin K, and diosbulbin L (*E*_dock_ = −111.2, −112.1, and −110.8 kJ/mol, respectively). Each of these ligands docked in the hydrophobic binding pocket with the furan group forming a hydrogen bond to the guanidine of Arg 394. The methyl ester of diosbulbin F and the carboxylate of diosbulbin L also formed hydrogen bonds to the imizadole of His 524. These three diterpenoids also docked strongly to ERβ. In addition, diosbulbin F, diosbulbin H, ptycho-6α,7α-diol, and ptycholide IV all had docking energies more exothermic (*E*_dock_ = −114.8, −114.1, −122.9, and −114.7 kJ/mol) than the co-crystallized ligand, 2-(5-hydroxynaphthalen-1-yl)-1,3-benzooxazol-6-ol (*E*_dock_ = −111.3 kJ/mol). Ptycho-6α,7α-diol docked with ERβ with hydrogen bonds between the lactone carbonyl of the ligand and Arg 346 and the 6-OH group of the ligand with His 475.

### Flavonoids

The structures of the flavonoid ligands examined in this work are shown in Figures 10, 11, 12, 13, 14, 15, 16, and 17, and the docking energies are listed in Table 6. Of the flavonoids, luteolin-8-propenoic acid docked the strongest to ERα, with a docking energy of −113.127 kJ/mol, more exothermic than those of estradiol, zearalenone, or genistein. A common docking orientation for phenolic ligands in ERα is the hydrophobic pocket of Leu 387, Phe 404, Met 388, and Leu 391, along with edge-to-face π–π interactions with Phe 404, and hydrogen bonds between the phenolic –OH group and the guanidine group of Arg 394 and the carboxylate of Glu 356. The 7-OH group of the ligand made an additional hydrogen bond with the carbonyl oxygen of Gly 521. No other flavonoid ligands showed notably strong docking with ERα.

Luteolin-8-propenoic acid was also the strongest docking flavonoid with ERβ (*E*_dock_ = −123.1 kJ/mol), is far more exothermic than estradiol, zearalenone, and the co-crystallized ligand, 2-(3-fluoro-4-hydroxyphenyl)-7-vinyl-1,3-benzoxazol-5-ol (*E*_dock_ = −106.2 kJ/mol). As observed in other phenolic ligands with ERβ, luteolin-8-propenoic acid occupied the hydrophobic pocket formed by Leu 298, Leu 339, and Phe 356; edge-to-face π–π interactions of the phenolic ligand with Phe 356 and hydrogen boding of the phenolic –OH group with the carbonyl group of Leu 339, the guanidine group of Arg 346, and the carboxylate of Glu 305. The 7-OH group of the ligand made additional hydrogen bonds with the carbonyl oxygen of Gly 472 and His 475. Casticin (*E*_dock_ = −106.4 kJ/mol), gonzalitosin (*E*_dock_ = −106.3 kJ/mol), gossypetin (*E*_dock_ = −105.7 kJ/mol), laricitrin (*E*_dock_ = −107.3 kJ/mol), myricetin (*E*_dock_ = −106.2 kJ/mol), quercetin (*E*_dock_ = −106.0 kJ/mol), santin (*E*_dock_ = −108.0 kJ/mol), and tricetin (*E*_dock_ = −106.2 kJ/mol), all had more exothermic docking energies than estradiol or zearalenone but were less exothermic than the co-crystallized ligand, 2-(5-hydroxynaphthalen-1-yl)-1,3-benzooxazol-6-ol (*E*_dock_ = −111.3 kJ/mol).

### Isoflavonoids

The docking energies of the isoflavonoids are summarized in Table 7 and the structures are shown in Figure 18, 19, and 20. Genistein is the quintessential estrogenic isoflavonoid, but it is a weaker docking ligand than estradiol or zearalenone for either ERα or ERβ. The strongest docking isoflavonoid with ERα was the aglycone of licoagroside A (*E*_dock_ = −100.5 kJ/mol), but this was weaker than zearalenone. On the other hand, several isoflavonoid ligands docked to ERβ more strongly than zearalenone: licoagroside A aglycone (*E*_dock_ = −107.7 kJ/mol), 1-methoxyphaseollin (*E*_dock_ = −110.5 kJ/mol), pratensein (*E*_dock_ = −106.4 kJ/mol), and 3′,5,7-trihydroxy-5′-methoxyisoflavone (*E*_dock_ = −107.9 kJ/mol), but none of these docked more strongly than the synthetic co-crystallized ligand, 2-(5-hydroxynaphthalen-1-yl)-1,3-benzooxazol-6-ol (*E*_dock_ = −111.3 kJ/mol).

### Lignans

The structures and the docking energies of the lignans are shown in Figure 21 and Table 8, respectively. Nortrachelogenin and 7′-hydroxymatairesinol were the strongest docking lignans to ERα (*E*_dock_ = −112.0 and −112.3 kJ/mol, respectively). Nortrachelogenin was also the strongest docking lignan to ERβ (*E*_dock_ = −125.4 kJ/mol). Sesamin showed notable selectivity for ERβ over ERα (*E*_dock_ = −121.8 and −99.1 kJ/mol, respectively). Nortrachelogenin occupied the same orientation and hydrogen-bonding pattern in both ERα and ERβ, with one of the phenolic –OH groups hydrogen bonded to argenine in the binding pocket (Arg 394 in ERα; Arg 346 in ERβ) and the other phenolic –OH group hydrogen bonded to the histidine (His 524 in ERα; His 475 in ERβ).

### Phenanthrenoids

The docking energies and structures of phenanthrenoids are shown in Table 9 and Figure 22, respectively. None of the phenanthrenoids examined in this work showed docking energies lower than estradiol or zearalenone for ERα or ERβ.

### Miscellaneous phenolics

The docking energies for miscellaneous herbal phenolic compounds are listed in Table 10, and the structures are shown in Figures 23, 24, and 25. The strongest docking ligands of the miscellaneous phenolic compounds for ERα was cimicifugic acid F (*E*_dock_ = −126.2 kJ/mol), and this ligand also docked strongly with ERβ (*E*_dock_ = −125.2 kJ/mol). Several other phenolic ligands docked with very exothermic energies to ERβ: the aglycone of agnucastoside C (*E*_dock_ = −130.0 kJ/mol), caffeoyl-*p*-coumaroyl tartaric acid (*E*_dock_ = −129.8 kJ/mol), cimiracemate B (*E*_dock_ = −127.3 kJ/mol), cimiracemate D (*E*_dock_ = −128.5 kJ/mol), and fukinolic acid (*E*_dock_ = −127.3 kJ/mol).

Analogous to other phenolic compounds (see above), the cinnamate moiety of the lowest-energy pose of cimicifugic acid F in ERα shows edge-to-face π–π interactions with Phe 404, and hydrogen bonding between the –OCH_3_ group and the guanidine group of Arg 394. Additionally, the 3-carboxylate group of the ligand is hydrogen-bonded to His 524, and the phenolic group fits into a hydrophobic pocket formed by Met 388, Met 421, and Ile 424.

The lowest-energy docked pose of the aglycone of agnucastoside C with ERβ, as with other phenolic compounds (see above), has the *p*-coumarate phenolic –OH group hydrogen bonded to the carbonyl group of Leu 339 and the guanidine group of Arg 346, and edge-to-face π–π interactions of the phenolic ligand with Phe 356. The cyclic hemiacetal group is hydrogen bonded to His 475.

### Sesquiterpenoids

Of the sesquiterpenoids, only cinnamoylechinadiol gave a notable docking energy (−120.8 kJ/mol) with ERβ (Figure 26, Table 11).

### Steroids

**Figure 48:**
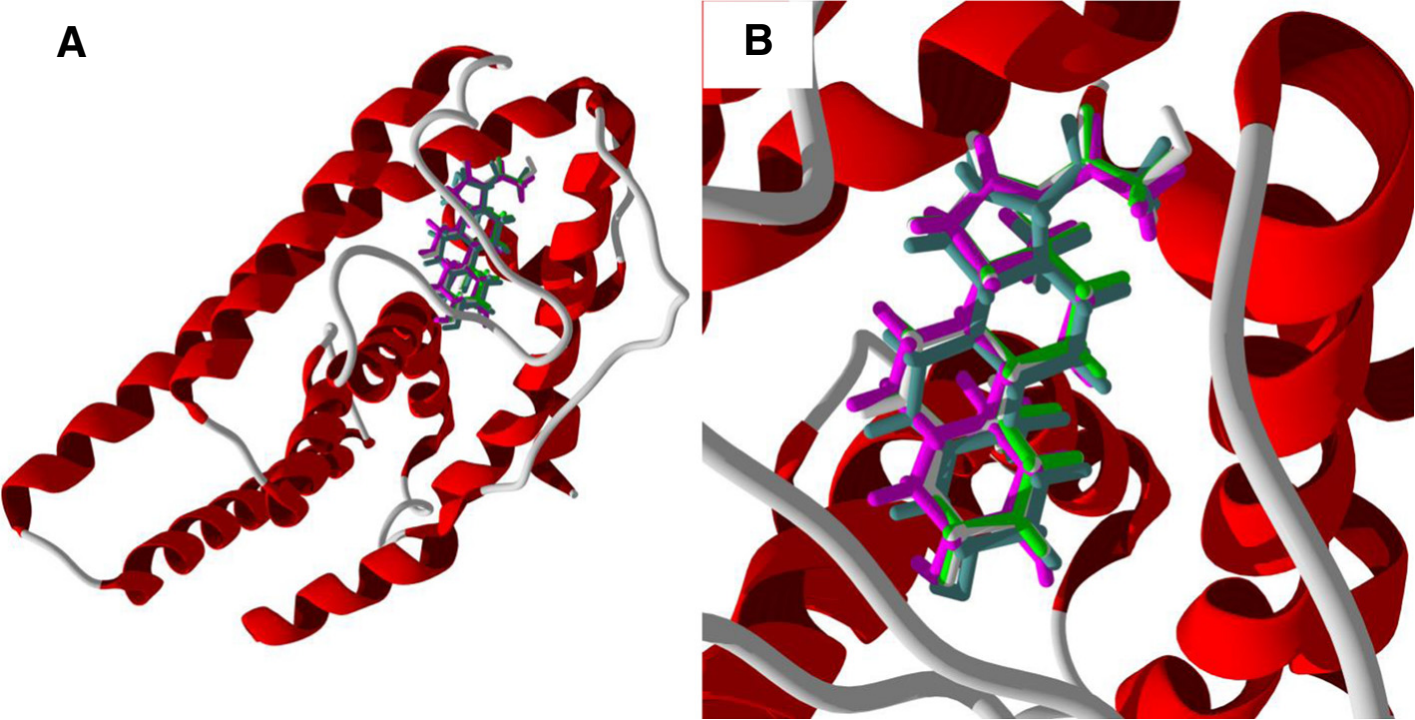
**Lowest-energy docked poses of pregnane steroids [2,3-dihydroxypregn-16-en-20-one (magenta), 3,16-dihydroxypregn-5-en-20-one (dark green), 3,21-dihydroxypregna-5,16-dien-20-one (white), and pregnadienolone (bright green)] with ERβ (PDB 1U3R). A:** Docked poses showing the entire ribbon structure of the protein. **B:** Close-up of the docked poses.

The steroidal ligands examined in this study are shown in Figures 27, 28, 29, 30, 31, 32, 33, and 34, and their docking energies are listed in Table 12. Several pregnane steroids exhibited docking energies less than the co-crystallized ligand 2-(5-hydroxynaphthalen-1-yl)-1,3-benzooxazol-6-ol (*E*_dock_ = −111.3 kJ/mol) for ERβ: 2,3-Dihydroxypregn-16-en-20-one (*E*_dock_ = −116.6 kJ/mol), 3,16-dihydroxypregn-5-en-20-one (*E*_dock_ = −116.6 kJ/mol), 3,21-dihydroxypregna-5,16-dien-20-one (*E*_dock_ = −121.4 kJ/mol), and pregnadienolone (*E*_dock_ = −115.4 kJ/mol). The lowest-energy docked poses of the pregnane ligands show them all to adopt the same orientation (Figure 48) with key hydrogen-bonding interactions of the 3-OH group of the steroids with the guanidine of Arg 346 and the amide carbonyl of Leu 339, and the 20-ketone group of the ligand with the imidazole N-H of His 475. Two ligands, 3,16-dihydroxypregn-5-en-20-one and 3,21-dihydroxypregna-5,16-dien-20-one, showed selectivity for ERβ over ERα (24.8 and 18.4 kJ/mol, respectively).

### Stilbenoids

Structures and docking energies for the stilbenoid ligands are shown in Figures 35 and 36, and Table 13, respectively. Several stilbenoid ligands showed notably strong docking energies; lower than estradiol, zearalenone, or the respective co-crystallized ligands: 3-acetoxy-4′,5-dihydroxy-3′-prenyldihydrostilbene (*E*_dock_ to ERα = −119.0 kJ/mol), licoagrodione (*E*_dock_ to ERβ = −116.9 kJ/mol), 3,3′,4,5′-tetrahydroxy-4′,5-diprenylbibenzyl (*E*_dock_ to ERβ = −117.1 kJ/mol), 3,3′,4,5′-tetrahydroxy-5-prenylbibenzyl (*E*_dock_ to ERβ = −115.0 kJ/mol), and uralstilbene (*E*_dock_ to ERβ = −122.1 kJ/mol). Prenylation of stilbenoids seems to improve docking energies by about 20 kJ/mol.

### Triterpenoids

Docking energies are presented in Table 14 and structures of triterpenoid ligands are illustrated in Figures 37, 38, 39, 40, and 41. Unlike the pregnane steroids, there were no triterpenoid ligands that showed good docking with either ERα or ERβ.

### Miscellaneous phytochemicals

Several miscellaneous phytochemicals found in herbal supplements were included in this study (Table 15, Figures 42 and 43). Of these ligands, orobanchyl acetate gave excellent docking energies for both ERα and ERβ (*E*_dock_ = −111.3 and −122.8 kJ/mol, respectively).

## Discussion

### *Angelica sinensis*

Dong quai (*Angelica sinensis* root) has been used in Chinese traditional medicine for thousands of years for various female health conditions (e.g., dysmenorrhea, pelvic pain, symptoms of menopause) (Chye 2006; Al-Bareeq et al. 2010; Fang et al. 2012). In spite of its history, dong quai provided no clinical relief of menopausal symptoms (Hirata et al. 1997). In fact, dong quai has been shown to stimulate the growth of MCF-7 (ER+ human mammary carcinoma) cells (Lau et al. 2005), but does not bind either ERα or ERβ (Liu et al. 2001). The plant contains several miscellaneous phytochemicals, only two of which have notable docking energies, 10-angeloylbutylphthalide (−107.1 kJ/mol with ERβ) and angeliferulate (−110.7 and −121.5 kJ/mol with ERα and ERβ, respectively).

### *Centella asiatica*

*Centella asiatica* (gotu kola) has been used in Ayurvedic traditional medicine for cognitive enhancement (Rao et al. 2005), to alleviate symptoms of anxiety and promote relaxation (Wijeweera et al. 2006), as well as for headache, body ache, asthma, ulcers, and wound healing (Kumar and Gupta 2002). Animal studies have revealed cognitive enhancement (Kumar and Gupta 2002; Rao et al. 2005), neuroprotective (Subathra et al. 2005), and anxiolytic (Wijeweera et al. 2006) effects. To our knowledge, there have been no reports on the estrogenic activity of *C. asiatica*.

The plant contains the flavonoids castillicetin, castilliferol, kaempferol, and quercetin; the triterpenoids 2,3,20,23-tetrahydroxy-28-ursanoic acid, 2,3,23-trihydroxy-20-ursen-28-oic acid, 2,3-dihydroxy-5-(hydroxymethyl)-24-norolean-12-en-28-oic acid, 3,6,23-trihydroxy-12-ursen-28-oic acid, 6β-hydroxymaslinic acid, asiatic acid, asiaticoside G, betulafolienetriol, centellasapogenol A, centelloside A, corosolic acid, isothankunic acid, madasiatic acid, madecassic acid, quasipanaxadiol, terminolic acid, uncargenin C and zemoside A; the steroid campesterol, and the miscellaneous compounds asiaticin, homosilphiperfoloic acid, and irbic acid. Both quercetin and asiaticin had notable exothermic docking energies with ERβ (−106.0 and −109.0 kJ/mol, respectively). Quercetin has shown preferential binding to ERβ (Kuiper et al. 1998).

### *Cimicifuga racemosa* (syn. *Actaea racemosa*)

Although black cohosh extracts have demonstrated clinical efficacy against some symptoms of menopause (Lieberman 1998; McKenna et al. 2001; Liske et al. 2002; Pockaj et al. 2004; Wuttke et al. 2006), several studies have demonstrated little or no estrogenic activity (Liu et al. 2001; Kronenberg 2003; Lupu et al. 2003; Mahady 2003). The efficacy of *C. racemosa* extracts on post-menopausal symptoms has been attributed to partial agonism of the serotonin receptor (Burdette et al. 2003) and the μ-opiate receptor (Rhyu et al. 2006).

*C. racemosa* extracts have revealed several triterpenoids (Shao et al. 2000), phenylpropanoids (Chen et al. 2002) and caffeic acid derivatives (Li et al. 2003). Very few of the *C. racemosa* triterpenoids showed negative docking energies and are, therefore, unlikely estrogen receptor binding agents. Several *C. racemosa* phenolic compounds did show remarkable docking affinities for both ERα and ERβ: cimicifugic acid A, cimicifugic acid B, cimicifugic acid G, cimiciphenol, cimiciphenone, cimiracemate A, cimiracemate B, cimiracemate C, cimiracemate D, and fukinolic acid. It is likely that any estrogenic activity of *C. racemosa* extract (Seidlová-Wuttke et al. 2003a) is due to the presence of phenolic components rather than triterpenoids.

### *Dioscorea villosa*

The rhizomes of wild yam, *Dioscorea villosa*, have been used to treat symptoms associated with menopause and premenstrual syndrome (PMS) as well as to relieve labor pains and sooth dysmenorrhea (Dutta 2015). The genus contains numerous steroidal glycosides (Sautour et al. 2006; Sautour et al. 2007; Ali et al. 2013). In this work, we have carried out *in-silico* screening of phytochemicals from the genus *Dioscorea* (Dictionary of Natural Products 2014). Of these, the diosbulbins D, F, H, J, K, and L (diterpenoids from *D. bulbifera* (Komori 1997; Liu et al. 2010) gave remarkable docking energies with both ERα and ERβ while the pregnane steroids 3,16-dihydroxypregn-5-en-20-one, 3,21-dihydroxypregna-5,16-dien-20-one, ergost-5-ene-3,26-diol, and pregnadienolone, showed selective docking with ERβ. Interestingly, neither furostane nor the spirostane steroids, common in *Dioscorea* spp. docked well with the estrogen receptors. It is worth noting that clinical studies have shown *D. villosa* to have little effect on menopausal symptoms (Komesaroff et al. 2001).

### *Echinacea* spp.

*Echinacea* (*E. angustifolia*, *E. pallida*, and *E. purpurea*) is one of the most popular herbal supplements sold in the United States and has been used as a treatment for the common cold, coughs, bronchitis, upper respiratory infections, and inflammatory conditions (Percival 2000). Recent studies have demonstrated *Echinacea* to exhibit immune-system-stimulating activity (Block and Mead 2003). Phytochemicals that have been isolated from *Echinacea* spp. include chicoric acid and monomethyl and dimethyl ethers, trichocarpinine, cinnamoylechinadiol, cinnamoylechinaxanthol, cinnamoylepoxyechinadiol, cinnamoyldihydroxynardol, caftaric acid, caffeoyl-*p*-coumaroyltartaric acid, burkinabin A, burkinabin B, kaempferol, luteolin, and quercetin. Although *Echinacea* has not shown estrogenic activity (Zava et al. 1998), six phytochemicals were identified in this docking study that showed strong docking to the estrogen receptor: the flavonoid quercetin; the phenolic compounds caffeoyl-*p*-coumaroyltartaric acid, caftaric acid, and chicoric acid; and the sesquiterpenoids cinnamoylechinadiol and cinnamoylepoxyechinadiol.

### *Gingko biloba*

*G. biloba* is commonly used as a supplement to improve cognitive abilities (Kennedy et al. 2000), and for women specifically, it has been used to treat some of the side effects accompanying menopause (Oh and Chung 2004). The extracts of *G. biloba* have previously been shown to exhibit feeble estrogenic effects, and act as selective estrogen receptor modulators (SERMs) with the α and β estrogen receptors (Oh and Chung 2006). Phytochemical analyses have revealed *G. biloba* extracts to contain the flavonoids (2*R*,3*S*,4*S*)-3,3′,4,4′,5,5′,7-heptahydroxyflavan, 8-(5-carboxy-2-methoxyphenyl)-5,7-dihydroxy-4′-methoxyflavone, acacetin, amentoflavone, apigenin, bilobetin, 5′-methoxybilobetin, epigallocatechin, ginkgetin, isoginkgetin, kaempferol, luteolin, quercetin, sciadopitysin, and tricetin, as well as the lignan sesamin, the sesquiterpenoids bilobanol, and the steroid globosterol. Of these, quercetin, tricetin, and sesamin gave large negative docking energies. Sesamin, in particular, docked strongly with ERβ.

### *Glycyrrhiza glabra*

The phytochemistry of *Glycyrrhiza glabra* has been well studied and numerous compounds have been isolated and identified, including aurones (licoagroaurone and licoagrone), chalcones (1,2-dihydroparatocarpin A, 2,4,4′-trihydroxychalcone, 4-hydroxychalcone, cordifolin, isoliquiriteginin, licoagrochalcones A-D, licochalcones A and B, α,2′,4,4′-tetrahydroxydihydrochalcone, and kanzonol Y), coumarins (2′-*O*-methylglabridin, 3,4-didehydroglabridin, 3′-hydroxy-4′-methoxyglabridin, 4′-*O*-methylgrabridin, 4′-*O*-methylkanzonol W, bergapten, gancaonin F, glabrene, glabrocoumarin, glycycoumarin, glyinflanin H, hispaglabridin A, hispaglabridin B, isoglycycoumarin, isoglycyrol, kanzonol U, kanzonol V, kanzonol W, and licocoumarin A), flavonoids (3-hydroxyglabrol, 6-prenyleriodictyol, 6-prenylpinocembrin, folerogenin, glabranin, glabrol, isolicoflavonol, isoschaftoside, isoviolanthin, kaempferol, kumatakenin, licoagrodin, licoflavanone, naringenin, norwogonin, pinocembrin, quercetin, shinflavanone, and vitexin), isoflavonoids (1-methoxyphaseollin, 2′,4′,5,7-tetrahydroxy-3′,8-diprenylisoflavanone, 7-acetoxy-2-methylisoflavone, 7-hydroxy-2-methylisoflavone, 7-methoxy-2-methylisoflavone, 8-prenylphaseollinisoflavan, genistein, glabraisoflavanone A, glabraisoflavanone B, glabroisoflavanone A, glabridin, glabroisoflavanone B, glabrone, glyasperin B, glyasperin K, glyzaglabrin, glyzarin, isoderrone, isoglabrone, isomucronulatol, kanzonol R, kanzonol T, kanzonol X, licoagroside A, licoricidin, lupiwighteone, phaseollinisoflavan, prunetin, shinpterocarpin, tetrapterol G, and wighteone), the lignan licoagrocarpin, stilbenoids (3,3′,4,5′-tetrahydroxy-4′,5-diprenylbibenzyl, 3,3′,4,5′-tetrahydroxy-5-prenylbibenzyl, 3,3′,5′-trihydroxy-4-methoxy-5-prenylbibenzyl, 3,3′,5′-trihydroxy-4-methoxybibenzyl, 3,4′,5-trihydroxy-3′,4-diprenylbibenzyl, 3,4′,5-trihydroxy-3′-prenyldihydrostilbene, 3-acetoxy,4′,5-dihydroxy-3′-prenyldihydrostilbene, licoagrodione, and uralstilbene), and triterpenoids (11-deoxoglycyrrhetic acid, 18α-glycyrrhetic acid, 18α-hydroxyglycyrrhetic acid, 21-hydroxyisoglabrolide, 24-hydroxyglycyrrhetic acid, 24-hydroxyliquiritic acid, 28-hydroxyglycyrrhetic acid, 3,24-dihydroxy-11,13(18)-oleanadien-30-oic acid methyl ester, 3,24-dihydroxy-9(11),12-oleanadien-30-oic acid, 9(11)-dehydroglycyrrhetic acid, β-amyrin, betulinic acid, desoxoglabrolide, glabric acid, glabrolide, glycyrrhetic acid, glycyrrhetol, isoglabrolide, lanosta-5,24-dien-3-ol, liquiridiolic acid, liquiritic acid, and liquoric acid).

Licorice (*Glycyrrhiza glabra*) root has been used for thousands of years by different cultures and for a variety of reasons (Fenwick et al. 1990). Although licorice root has been suggested as a treatment for symptoms of menopause (Ojeda 2003), *G. glabra* root extracts have been shown to be inactive in terms of ERα or ERβ binding (Liu et al. 2001). Nevertheless, however, fractionation of *G. glabra* extracts has revealed several ER-modulating components (Khalaf et al. 2010; Simons et al. 2011). Glabrene binds to human ER and shows estrogenic activity (Tamir et al. 2001; Simons et al. 2011). Our *in-silico* docking study shows glabrene to be a strongly docking ligand to both ERα and ERβ (−104.8 and −114.9 kJ/mol, respectively). Glabridin, on the other hand, displayed ERα-selective antagonism (Simons et al. 2011), in contrast to the docking results that showed glabridin to have ERβ docking selectivity (−15.8 and −92.9 kJ/mol, respectively). The chalcones isoliquiritigenin (Tamir et al. 2001; Maggiolini et al. 2002) and licochalcone A (Rafi et al. 2000), the flavonoid quercetin (Kuiper et al. 1998), and the isoflavonoid genistein (Ososki and Kennelly 2003) also bind to human ERα and show estrogenic activity. These ligands all show negative docking energies with ERα (range from −93.2 to −99.9 kJ/mol) and ERβ (−98.9 to −107.8 kJ/mol).

### *Lepidium meyenii*

Maca (*Lepidium meyenii*) is native to the central Andes of Peru (3500–4500 m asl) (Wang et al. 2007). The root has been used by native Amerindians to improve fertility, as an aphrodisiac for both men and women. Maca was found to increase sperm counts and gonadal mass in a rat model (Chung et al. 2005), to improve copulatory performance of male mice and rats (Zheng et al. 2000; Cicero et al. 2001), and to increase litter size (Ruiz-Luna et al. 2005) and pregnancy rates in female mice (Kuo et al. 2003). In adult human males, maca treatment led to increased semen volume and sperm count (Gonzales et al. 2001) and increased sexual desire (Gonzales et al. 2002; Stone et al. 2009). In addition, maca reduced sexual dysfunction in postmenopausal women (Brooks et al. 2008) and inhibited estrogen-deficient osteoporosis in ovariectomized rats (Zhang et al. 2006), but maca extracts have not shown estrogenic activity (Brooks et al. 2008). None of the *L. meyenii* phytochemicals investigated in this *in-silico* study showed remarkable docking energies, consistent with the non-estrogenic activity previously reported.

### *Ptychopetalum olacoides*, *P. uncinatum*

Muira puama (bark and root extracts of *P. olacoides* or *P. uncinatum*) has been used in Amazonian Brazil during highly stressful periods, to treat CNS-related ailments, neuromuscular problems, “nervous weakness”, sexual debility, frigidity, impotence, and rheumatism (Schultes and Raffauf 1990; Siqueira et al. 1998; Duke et al. 2009). Consistent with these traditional uses, *P. olacoides* ethanol root extract has shown memory retrieval improvement in young and aging mice (da Silva et al. 2004), *in-vitro* acetylcholine esterase inhibitory activity (Siqueira et al. 2003), and prevention of stress-induced hypothalamic-pituitary-adrenal hyperactivity (Piato et al. 2008). In addition, Muira puama formulations have demonstrated efficacy in treating male erectile dysfunction and low libido (Waynberg 1994) and low sex drive in women (Waynberg and Brewer 2000). A number of clerodane diterpenoids have been isolated from *P. olacoides* bark (Tang et al. 2008; Tang et al. 2009; Tang et al. 2011). Several of these have given excellent docking energies with the estrogen receptor, but two in particular, ptycho-6α,7α-diol and ptycholide IV had remarkable docking to ERβ (−122.9 and −114.7 kJ/mol, respectively). To our knowledge, the estrogenic effects of Muira puama have not been investigated.

### *Rhodiola rosea*

*R. rosea* is reputed to strengthen the nervous system, fight depression, enhance memory, and improve energy levels (Brown et al. 2002), which has been attributed to adaptogenic properties of the herb (Spasov et al. 2000; Darbinyan et al. 2000). The flavonoids gossypetin, herbacetin, and rhodiolin, and the lignan (+)-lariciresinol, have been identified in *R. rosea*. Lariciresinol showed strong docking to both ERα and ERβ. There are conflicting reports on the potential estrogenic effects of *R. rosea*, however (Eagon et al. 2004; Kim et al. 2005).

### *Sambucus nigra*

The bark, leaves, flowers, fruit, and root extracts of black elderberry (*Sambucus nigra*) have been used traditionally to treat respiratory ailments such as bronchitis, cough, influenza, and upper respiratory infections (Zakay-Rones et al. 1995; Krawitz et al. 2011). Compounds identified in *S. nigra* extracts include α-amyrin, β-amyrin, α-amyrone, betulin, campesterol, cycloartenol, lupeol, oleanolic acid, quercetin, ursolic acid, and dihydrodehydrodiconiferyl alcohol (9-acetate). Of these, only the flavonoid quercetin showed good docking to the estrogen receptor. To our knowledge, there are no reports on the estrogenic activity of *S. nigra* extracts.

### *Silybum marianum*

Milk thistle, *Silybum marianum*, extracts (silymarin) have been used for centuries to treat liver diseases (Flora et al. 1998). Silymarin contains the flavonoids apigenin, chrysoeriol, cisilandrin, eriodictyol, isocisilandrin, isosilandrin A, isosilandrin B, isosilybin A, isosilybin B, isosilybin C, isosilybin D, isosilychristin, kaempferol, naringenin, neosilyhermin A, neosilyhermin B, quercetin, silandrin A, silandrin B, silyamandin, silybin A, silybin B 2,3-dehydrosilybin, silychristin, 2,3-dehydrosilychristin, silychristin B, silydianin, silyhermin, silymonin, taxifolin; the lignan (*Z*)-dehydrodiconiferyl alcohol; the steroids 24-methylenelanost-8-ene-3,25,28-triol and marianine; and the triterpenoids silymin A and silymin B. Silybin B (also called silibinin) has been shown to selectively bind to the ERβ receptor rather than ERα (Seidlová-Wuttke et al. 2003b) and previous docking studies have shown selective docking to ERβ over ERα (El-Shitany et al. 2010). In contrast, our current docking study revealed that neither silybin A nor silybin B gave negative docking energies with either ERα or ERα. Quercetin and taxifolin, on the other hand, gave docking energies comparable to zearalenone with ERβ (−106.0, −104.6, and −104.5 kJ/mol, respectively), and these flavonoids have also shown estrogenic activity (Plíšková et al. 2005). Silymarin modulation of ERβ may be responsible for the estrogenic effects of the extract (Seidlová-Wuttke et al. 2003b; Plíšková et al. 2005; El-Shitany et al. 2010).

### *Tribulus terrestris*

*Tribulus terrestris* has been used to contribute to physical and sexual strength (De Combarieu et al. 2003; Neychev and Mitev 2005). The plant is rich in steroidal glycosides (Wu et al. 1996; Yan et al. 1996; De Combarieu et al. 2003; Dinchev et al. 2008) and *T. terrestris* extracts have shown androgenic effects in animal models (Gauthaman et al. 2002; Gauthaman and Ganesan 2008), but had no influence on androgen production (Neychev and Mitev 2005) or gains in strength or muscle mass (Rogerson et al. 2007) in young men. To our knowledge, there have been no reports on the estrogenic effects of *T. terrestris*.

In addition to steroids, several alkaloids (*N-trans*-feruloyltyramine, perlolyrine, terresoxazine, terrestriamide, tribulusamide A and tribulusamide B) and flavonoids (isorhamnetin, kaempferol, and quercetin) have been found in *T. terrestris. In-silico* molecular docking has shown that *N-trans*-feruloyltyramine docks strongly to both ERα and ERβ, perlolyrine docks strongly to ERβ, terrestribisamide docks strongly to ERα, quercetin docks strongly to ERβ, and the steroid 2,3-dihydroxypregn-16-en-20-one docks strongly to both ERα and ERβ.

### *Trifolium pratense*

The alkaloids *cis-* and *trans-*clovamide, several coumarins, flavonoids, and isoflavonoids have been identified in red clover (*Trifolium pratense*). Although there have been no ethnobotanical reports to support it, the presence of isoflavones has led to suggest that red clover may serve as a phytochemical alternative to post-menopausal hormone replacement therapy (Coon et al. 2007). Red clover extract has been shown to exhibit weakly estrogenic effects in a rat model (Burdette et al. 2002) and does show *in-vitro* ERα and ERβ binding ability (Dornstauder et al. 2001; Beck et al. 2003; Overk et al. 2005). The clinical effectiveness of red clover has not, however, been demonstrated (Fugh-Berman and Kronenberg 2001; Booth et al. 2006).

Biochanin A, daidzein, formononetin, and genistein have been identified as *T. pratense* isoflavonoids with ERα and ERβ binding activity (Beck et al. 2003; Pfitscher et al. 2008) and these compounds did show a binding preference for ERβ. Molecular docking of these ligands also showed preference for ERβ. They were not, however, the best docking ligands of *T. pratense* phytochemicals. The alkaloids *cis-* and *trans-*clovamide, and the coumarin pratenol B showed good docking to both ERα and ERβ. Of the *T. pratense* isoflavonoids, calycosin and pseudobaptigenin had more exothermic docking energies to ERβ than did biochanin A, daidzein, formononetin, or genistein.

### *Trigonella foenum-graecum*

Fenugreek (*Trigonella foenum-graecum*) is used as an antidiabetic (Abdel-Barry et al. 1997) and for lowering blood lipid and cholesterol levels (Prasanna 2000). Phytochemicals identified in *T. foenum-graecum* include the alkaloid gentianine; the coumarins 7-acetoxy-4-methylcoumarin, trigocoumarin, and trigoforin; the flavonoids 2″-*O-p*-coumaroylvitexin, 2″-*O-p-*coumaroylorientin, 6,8-digalactosylapigenin, 8-galactopyranosyl-6-quinovopyranosylapigenin, 8-β-D-galactopyranosyl-6-β-D-xylopyranosylapigenin, isoorientin, isovitexin, kaempferol, luteolin, neocorymboside, orientin, quercetin, trigraecum, vicenin 1, vicenin 2, and vicenin 3; the isoflavonoid 3′,5,7-trihydroxy-5′-methoxyisoflavone; *p*-coumaric acid; and the steroids 25*R*-spirosta-3,5-diene (= Δ^3,5^-deoxyneotigogenin), diosgenin, furostane-2,3,22,26-tetrol (= trigoneoside aglycone), gitogenin, neotigogenin, tigogenin, and yamogenin. Quercetin and 3′,5,7-trihydroxy-5′-methoxyisoflavone were the strongest docking ligands for ERβ. Fenugreek seed extract has shown estrogenic activity (Sreeja et al. 2010).

### *Turnera aphrodisiaca* (syn. *T. diffusa*)

Damiana leaf has been used in traditional medicine in Neotropical cultures as a stimulant and aphrodisiac (Morton 1981), and the herb is marketed as a sexual enhancer for men and women. Extracts of *T. aphrodisiaca* have shown aphrodisiac activity in mouse (Helmrick and Reiser 2000; Kumar et al. 2009) and rat (Estrada-Reyes et al. 2009; Estrada-Reyes et al. 2013) models, as well as anxiolytic activity in mice (Kumar and Sharma 2005). Phytochemical investigations have revealed damiana to be rich in flavonoids, including acacetin, apigenin, gonzalitosin, laricitrin, luteolin-8-propenoic acid, orientin, orientin-3″-ketone, pinocembrin, and syringetin (Piacente et al. 2002; Zhao et al. 2007). The extract has shown anti-aromatase (due primarily to acacetin and pinocembrin) and estrogenic activity (due primarily to apigenin, *Z*-echinacin, and pinocembrin) (Zhao et al. 2008). In contrast to these experimental results, molecular docking revealed the strongest docking *Turnera* compound to be luteolin-8-propenoic acid (−113.1, −123.1 kJ/mol for ERα and ERβ, respectively) but this compound was inactive in the aromatase and estrogen assays. In contrast, pinocembrin, which was active in both experimental assays, had ERα and ERβ docking energies of −81.4 and −87.9 kJ/mol, respectively.

### *Vitex agnus-castus*

*Vitex agnus-castus*, “chaste tree”, has been used as a tonic for female reproductive disorders, including menstrual disorders (amenorrhea, dysmenorrhea), premenstrual syndrome (PMS, corpus luteum insufficiency, hyperprolactinemia, infertility, menopause, and disrupted lactation (Daniele et al. 2005; van Die et al. 2013). *V. agnus-castus* extracts contain several flavonoids (casticin, isoorientin, 6″-caffeoylisoorientin, 6″-caffeoylisoorientin-4″-methyl ether, isovitexin, luteolin, orientin, santin, 5-*O*-demethyltangeretin, and vitexin), diterpenoids (8,14-labdadiene-6,7,13-triol-6,7-diacetate, viteagnuside A, viteagnusin A, viteagnusin B, viteagnusin D, viteagnusin E, viteagnusin F, viteagnusin G, viteagnusin H, viteagnusin I, viteagnusin J, and vitexlactam A), the isoflavonoid vitexcarpan, and the phenolic compounds agnucastoside C and agnuside. Based upon docking energies with ERβ, casticin, santin, vitexcarpan, and the aglycones of agnucastoside C and agnuside, could be expected to exhibit ER modulation. Indeed, *V. agnus-castus* extracts have been shown to bind to ERα (Liu et al. 2001) and ERβ (Jarry et al. 2003). It has been suggested that *V. agnus-castus* is a source of 3-ketosteroids with progesterone-like activity (Bruneton 1999; Dweck 2006).

## Conclusions

This molecular docking study has revealed that almost all popular herbal supplements contain phytochemical components that may bind to the human estrogen receptor and exhibit selective estrogen receptor modulation. As such, these herbal supplements may cause unwanted side effects related to estrogenic activity. For example, estrogenic agents may be effective and potent growth stimulators of estrogen-receptor positive tumors and pose a hazard to patients with breast cancer who have ER-positive tumors and who are being treated with antiestrogens.

The strongest docking (most exothermic docking energies) phytochemical ligands were phenolic compounds and the weakest docking ligands were triterpenoids. A common binding motif for phenolic ligands in ERα is the hydrophobic pocket of Leu 387, Phe 404, Met 388, and Leu 391, along with edge-to-face π–π interactions with Phe 404, and hydrogen bonds between the phenolic –OH group and the guanidine group of Arg 394 and the carboxylate of Glu 356. Similarly, interactions of phenolic ligands with ERβ include binding in a hydrophobic pocket formed by Leu 298, Leu 339, and Phe 356; edge-to-face π–π interactions of the phenolic ligand with Phe 356 and hydrogen boding of the phenolic –OH group with the carbonyl group of Leu 339, the guanidine group of Arg 346, and the carboxylate of Glu 305. Common hydrogen-bonding residues in the binding sites of ERα are the guanidine group of Arg 394, the imidazole group of His 524. Hydrogen-bonding residues of ERβ are Arg 346, His 475.

There are several limitations to these docking results:

- Some of the herbal phytochemicals examined may not be bioavailable due to limited solubility, membrane permeability;
- This docking study has only examined docking of the natural ligands (or their aglycones) and does not take into account *in-vivo* hydrolysis or other metabolic derivatization;
- The docking studies do not account for synergism in the estrogen receptor binding;
- The molecular docking method itself suffers from inherent limitations (e.g., the protein is modeled as a rigid structure without flexibility, solvation of the binding site and the ligand is excluded, and free-energy estimation of protein-ligand complexes is largely ignored) (Yuriev et al. 2011; Yuriev and Ramsland 2013).
- Docking energies do not provide information about whether strongly binding ligands may function as agonists or antagonists of the estrogen receptor.

## Abbreviations

ERα: Estrogen receptor α
ERβ: Estrogen receptor β
ER+: Estrogen receptor positive
MMFF: Merck molecular force field
PDB: Protein data bank
PMS: Pre-menstrual syndrom
RMSD: Root mean square deviation
SERM: Selective estrogen receptor modulator

## References

[CR1] Abdel-Barry JA, Abdel-Hassan IA, Al-Hakiem MHH (1997). Hypoglycaemic and antihyperglycaemic effects of *Trigonella foenum-graecum* leaf in normal and alloxan induced diabetic rats. J Ethnopharmacol.

[CR2] Al-Bareeq RJ, Ray AA, Nott L, Pautler SE, Razvi H (2010). Dong quai (*Angelica sinensis*) in the treatment of hot flashes for men on androgen deprivation therapy: results of a randomized double-blind placebo controlled trial. Can Urol Assoc J.

[CR3] Ali Z, Smillie TJ, Khan IA (2013). Cholestane steroid glycosides from the rhizomes of *Dioscorea villosa*. Carbohydr Res.

[CR4] Au AM, Ko R, Boo FO, Hsu R, Perez G, Yang Z (2000). Screening methods for drugs and heavy metals in Chinese patent medicines. Bull Environ Contam Toxicol.

[CR5] Beck V, Unterrieder E, Krenn L, Kubelka W, Jungbauer A (2003). Comparison of hormonal activity (estrogen, androgen, and progestin) of standardized plant extracts for large scale use in hormone replacement therapy. Mol Biol.

[CR6] Block KI, Mead MN (2003). Immune system effects of echinacea, ginseng, and astragalus: a review. Integr Cancer Ther.

[CR7] Booth NL, Piersen CE, Banuvar S, Geller SE, Shulman LP, Farnsworth NR (2006). Clinical studies of red clover (*Trifolium pratense*) dietary supplements in menopause: a literature review. Menopause.

[CR8] Brooks NA, Wilcox G, Walker KZ, Ashton JF, Cox MB, Stojanovska L (2008). Beneficial effects of *Lepidium meyenii* (Maca) on psychological symptoms and measures of sexual dysfunction in postmenopausal women are not related to estrogen or androgen content. Menopause.

[CR9] Brown RP, Gerbarg PL, Ramazanov Z (2002). *Rhodiola rosea* A phytomedicinal overview. Herbal Gram.

[CR10] Bruneton J (1999). Pharmacognosy Phytochemistry Medicinal Plants.

[CR11] Burdette JE, Liu J, Lantvit D, Lim E, Booth N, Bhat KPL, Hedayat S, Van Breemen RB, Constantinou AI, Pezzuto JM, Farnsworth NR, Bolton JL (2002). *Trifolium pratense* (red clover) exhibits estrogenic effects in vivo in ovariectomized Sprague–Dawley rats. J Nutr.

[CR12] Burdette JE, Liu J, Chen SN, Fabricant DS, Piersen CE, Barker EL, Pezzuto JM, Mesecar A, van Breemen RB, Farnsworth NR, Bolton JL (2003). Black cohosh acts as a mixed competitive ligand and partial agonist of the serotonin receptor. J Agric Food Chem.

[CR13] Calixto JB (2000). Efficacy, safety, quality control, marketing and regulatory guidelines for herbal medicines (phytotherapeutic agents). Braz J Med Biol Res.

[CR14] Chen SN, Fabricant DS, Lu ZZ, Zhang H, Fong HHS, Farnsworth NR (2002). Cimiracemates A-D, phenylpropanoids esters from the rhizomes of *Cimicifuga racemosa*. Phytochemistry.

[CR15] Chung F, Rubio J, Gonzales C, Gasco M, Gonzales GF (2005). Dose–response effects of *Lepidium meyenii* (Maca) aqueous extract on testicular function and weight of different organs in adult rats. J Ethnopharmacol.

[CR16] Chye PLH (2006). Traditional Asian folklore medicines in sexual health. Indian J Urol.

[CR17] Cicero AFG, Bandieri E, Arletti R (2001). *Lepidium meyenii* Walp. improves sexual behavior in male rats independently from its action on spontaneous locomotor activity. J Ethnopharmacol.

[CR18] Coon JT, Pittler MH, Ernst E (2007). *Trifolium pratense* isoflavones in the treatment of menopausal hot flushes: a systematic review and meta-analysis. Phytomedicine.

[CR19] Cupp MJ (1999). Herbal remedies: adverse effects and drug interactions. Am Fam Physician.

[CR20] da Silva AL, Piato ALS, Bardini S, Netto CA, Nunes DS, Elisabetsky E (2004). Memory retrieval improvement by *Ptychopetalum olacoides* in young and aging mice. J Ethnopharmacol.

[CR21] Daniele C, Thompson Coon J, Pittler MH, Ernst E (2005). Vitex agnus castus. Drug Saf.

[CR22] Darbinyan V, Kteyan A, Panossian A, Gabrielian E, Wikman G, Wagner H (2000). *Rhodiola rosea* in stress induced fatigue – A double blind cross-over study of a standardized extract SHR-5 with a repeated low-dose regimen on the mental performance of healthy physicians during night duty. Phytomedicine.

[CR23] De Combarieu E, Fuzzati N, Lovati M, Mercalli E (2003). Furostanol saponins from *Tribulus terrestris*. Fitoterapia.

[CR24] Dictionary of Natural Products on DVD v.23:1 (2014) CRC Press, Boca Raton, Florida, USA

[CR25] Dinchev D, Janda B, Evstatieva L, Oleszek W, Aslani MR, Kostova I (2008). Distribution of steroidal saponins in *Tribulus terrestris* from different geographical regions. Phytochemistry.

[CR26] Dornstauder E, Jisa E, Unterrieder I, Krenn L, Kubelka W, Jungbauer A (2001). Estrogenic activity of two standardized red clover extracts (Menoflavon®) intended for large scale use in hormone replacement therapy. J Steroid Biochem Mol Biol.

[CR27] Duke J, Bogenschutz MJ (1998) Dr. Duke’s phytochemical and ethnobotanical databases. http://www.ars-grin.gov/duke/

[CR28] Duke JA, Bogenschutz-Godwin MJ, Ottesen AR (2009). Duke’s Handbook of Medicinal Plants of Latin America.

[CR29] Dutta B (2015). Food and medicinal values of certain species of *Dioscorea* with special reference to Assam. J Pharmacog Phytochem.

[CR30] Dweck AC (2006). Isoflavones, phytohormones and phytosterols. J Appl Costetol.

[CR31] Eagon PK, Elm MS, Gerbarg PL, Brown RP, Check JJ, Diorio GJ, Houghton F (2004). Evaluation of the medicinal botanical *Rhodiola rosea* for estrogenicity. Proc Am Assoc Cancer Res.

[CR32] El-Shitany NA, Hegazy S, El-desoky K (2010). Evidences for antiosteoporotic and selective estrogen receptor modulator activity of silymarin compared with ethinyl estradiol in ovariectomized rats. Phytomedicine.

[CR33] Estrada-Reyes R, Ortiz-López P, Gutiérrez-Ortíz J, Martínez-Mota L (2009). *Turnera diffusa* Wild (Turneraceae) recovers sexual behavior in sexually exhausted males. J Ethnopharmacol.

[CR34] Estrada-Reyes R, Carro-Juárez M, Martínez-Mota L (2013). Pro-sexual effects of *Turnera diffusa* Wild (Turneraceae) in male rats involves the nitric oxide pathway. J Ethnopharmacol.

[CR35] Fang L, Xiao XF, Liu CX, He X (2012). Recent advance in studies on *Angelica sinensis*. Chinese Herbal Med.

[CR36] Fenwick GR, Lutomski J, Nieman C (1990). Liquorice, *Glycyrrhiza glabra* L. – Composition, uses and analysis. Food Chem.

[CR37] Flora K, Hahn M, Rosen H, Benner K (1998). Milk thistle (*Silybum marianum*) for the therapy of liver disease. Am J Gastroenterol.

[CR38] Fugh-Berman A, Kronenberg F (2001). Red clover (*Trifolium pratense*) for menopausal women: current state of knowledge. Menopause.

[CR39] Gauthaman K, Ganesan AP (2008). The hormonal effects of *Tribulus terrestris* and its role in the management of male erectile dysfunction – an evaluation using primates, rabbit and rat. Phytomedicine.

[CR40] Gauthaman K, Adaikan PG, Prasad RNV (2002). Aphrodisiac properties of *Tribulus terrestris* extract (Protodioscin) in normal and castrated rats. Life Sci.

[CR41] Gonzales GF, Córdova A, Gonzales C, Chung A, Vega K, Villena A (2001). *Lepidium meyenii* (maca) improved semen parameters in adult men. Asian J Androl.

[CR42] Gonzales GF, Córdova A, Vega K, Chung A, Villena A, Góñez C, Castillo S (2002). Effect of *Lepidium meyenii* (MACA) on sexual desire and its absent relationship with serum testosterone levels in adult healthy men. Andrologia.

[CR43] Halgren TA (1996). Merck molecular force field. I. Basis, form, scope, parameterization, and performance of MMFF 94. J Comput Chem.

[CR44] Helmrick L, Reiser C (2000). Aphrodisiac properties of *Turnera diffusa*. J Undergrad Res.

[CR45] Hirata JD, Swiersz LM, Zell B, Small R, Ettinger B (1997). Does dong quai have estrogenic effects in postmenopausal women? A double-blind, placebo-controlled trial. Fertil Steril.

[CR46] Jarry H, Spengler B, Porzel A, Schmidt J, Wuttke W, Christoffel V (2003). Evidence for estrogen receptor β-selective activity of *Vitex agnus-castus* and isolated flavones. Planta Med.

[CR47] Kennedy DO, Scholey AB, Wesnes KA (2000). The dose-dependent cognitive effects of acute administration of *Ginkgo biloba* to healthy young volunteers. Psychopharmacology (Berl).

[CR48] Khalaf I, Vlase L, Lazăr D, Corciovă A, Ivãnescu B, Lazăr MI (2010). HPLC-MS study of phytoestrogens from *Glycyrrhiza glabra*. Farmacia.

[CR49] Kim JB, Price RK, Stein RC, O’Hara MJ (2005). Risks of the complementary medicine *Rhodiola rosea* in breast cancer. J Nutr (Suppl).

[CR50] Komesaroff PA, Black CVS, Cable V, Sudhir K (2001). Effects of wild yam extract on menopausal symptoms, lipids and sex hormones in healthy menopausal women. Climacteric.

[CR51] Komori T (1997). Glycosides from *Dioscorea bulbifera*. Toxicon.

[CR52] Krawitz C, Abu Mraheil M, Stein M, Imirzalioglu C, Domann E, Pleschka S, Hain T (2011). Inhibitory activity of a standardized elderberry liquid extract against clinically-relevant human respiratory bacterial pathogens and influenza A and B viruses. BMC Complement Altern Med.

[CR53] Kretzschmar G, Zierau O, Wober J, Tischer S, Metz P, Vollmer G (2010). Prenylation has a compound specific effect on the estrogenicity of naringenin and genistein. J Steroid Biochem Mol Biol.

[CR54] Kronenberg F (2003). Black cohosh, a menopausal remedy, does not have estrogenic activity and does not promote breast cancer cell growth. Int J Oncol.

[CR55] Kuiper GGJM, Lemmen JG, Carlsson B, Corton JC, Safe SH, van der Saag PT, van der Burg B, Gustafsson JÅ (1998). Interaction of estrogenic chemicals and phytoestrogens with estrogen receptor β. Endocrinology.

[CR56] Kumar MHV, Gupta YK (2002). Effect of different extracts of *Centella asiatica* on cognition and markers of oxidative stress in rats. J Ethnopharmacol.

[CR57] Kumar S, Sharma A (2005). Anti-anxiety activity studies on homeopathic formulations of *Turnera aphrodisiaca* Ward. Evid Based Complement Alternat Med.

[CR58] Kumar S, Madaan R, Sharma A (2009). Evaluation of aphrodisiac activity of *Turnera aphrodisiaca*. Int J Pharmacog Phytochem Res.

[CR59] Kuo TF, Chang MH, Liau MY (2003). Effects of *Lepidium meyenii* Walp. (maca) on fecundity and puppy growth in mice. Taiwan Vet J.

[CR60] Lau CB, Ho TC, Chan TW, Kim SC (2005). Use of dong quai (Angelica sinensis) to treat peri- or postmenopausal symptoms in women with breast cancer: is it appropriate?. Menopause.

[CR61] Li W, Sun Y, Liang W, Fitzloff JF, van Breeman RB (2003). Identification of caffeic acid derivatives in *Actaea racemosa* (*Cimicifuga racemosa*, black cohosh) by liquid chromatography / tandem mass spectrometry. Rapid Commun Mass Spectrom.

[CR62] Lieberman S (1998). A review of the effectiveness of *Cimicifuga racemosa* (black cohosh) for the symptoms of menopause. J Womens Health.

[CR63] Liske E, Hänggi W, Henneicke-von Zepelin HH, Boblitz N, Wüstenberg P, Rahlfs VW (2002). Physiological investigation of a unique extract of black cohosh (*Cimicifugae racemosae rhozoma*): A 6-month clinical study demonstrates no systemic estrogeni effect. J Womens Health Gend Based Med.

[CR64] Liu J, Burdette JE, Xu H, Gu C, van Breemen RB, Bhat KPL, Booth N, Constantinou AI, Pezzuto JM, Fong HHS, Farnsworth NR, Bolton JL (2001). Evaluation of estrogenic activity of plant extracts for the potential treatment of menopausal symptoms. J Agric Food Chem.

[CR65] Liu H, Chou GX, Guo YL, Ji LL, Wang JM, Wang ZT (2010). Norclerodane diterpenoids from rhizomes of *Dioscorea bulbifera*. Phytochemistry.

[CR66] Lupu R, Mehmi I, Atlas E, Tsai MS, Pisha E, Oketch-Rabah HA, Nuntanakorn P, Kennelly EJ, Kronenberg F (2003). Black cohosh, a menopausal remedy, does not have estrogenic activity and does not promote breast cancer cell growth. Int J Oncol.

[CR67] Maggiolini M, Statti G, Vivacqua A, Gabriele S, Rago V, Loizzo M, Menichini F, Amdò S (2002). Estrogenic and antiproliferative activities of isoliquiritigenin in MCF7 breast cancer cells. J Steroid Biochem Mol Biol.

[CR68] Mahady GB (2003). Is black cohosh estrogenic?. Nutr Rev.

[CR69] Malamas MS, Manas ES, McDevitt RE, Gunawan I, Xu ZB, Collini MD, Miller CP, Dinh T, Henderson RA, Keith JC, Harris HA (2004). Design and synthesis of aryl diphenolic azoles as potent and selective estrogen receptor-beta ligands. J Med Chem.

[CR70] Manas ES, Unwalla RJ, Xu ZB, Malamas MS, Miller CP, Harris HA, Hsiao C, Akopian T, Hum WT, Malakian K, Wolfrom S, Bapat A, Bhat RA, Stahl ML, Somers WS, Alvarez JC (2004). Structure-based design of estrogen receptor-beta selective ligands. J Am Chem Soc.

[CR71] Manas ES, Xu ZB, Unwalla RJ, Somers WS (2004). Understanding the selectivity of genistein for human estrogen receptor-beta using X-ray crystallography and computational methods. Structure.

[CR72] McKenna DJ, Jones K, Humphrey S, Hughes K (2001). Black cohosh: efficacy, safety, and use in clinical and preclinical applications. Altern Ther Health Med.

[CR73] Molegro Virtual Docker version 6.0.0 (2013) Molegro ApS, Aarhus, Denmark

[CR74] Morton JF (1981). Atlas of Medicinal Plants of Middle America. Vol I.

[CR75] Neychev VK, Mitev VI (2005). The aphrodisiac herb *Tribulus terrestris* does not influence the androgen production in young men. J Ethnopharmacol.

[CR76] Oh SM, Chung KH (2004). Estrogenic effects of *Ginkgo biloba* extracts. Life Sci.

[CR77] Oh SM, Chung KH (2006). Antiestrogenic activities of *Ginkgo biloba* extracts. J Steroid Biochem Mol Biol.

[CR78] Ojeda L (2003). Menopause without Medicine.

[CR79] Ososki AL, Kennelly EJ (2003). Phytoestrogens: a review of the present state of research. Phytother Res.

[CR80] Overk CR, Yao P, Chadwick LR, Nikolic D, Sun Y, Cuendet MA, Deng Y, Hedayat AS, Pauli GF, Farnsworth NR, van Breemen RB, Bolton JL (2005). Comparison of the in vitro estrogenic activities of compounds from hops (*Humulus lupulus*) and red clover (*Trifolium pratense*). J Agric Food Chem.

[CR81] Percival SS (2000). Use of Echinacea in medicine. Biochem Pharmacol.

[CR82] Pfitscher A, Reiter E, Jungbauer A (2008). Receptor binding and transactivation activities of red clover isoflavones and their metabolites. J Steroid Biochem Mol Biol.

[CR83] Piacente S, Camargo EES, Zampelli A, Gracioso JS, Souza Brito AR, Pizza C, Vilegas W (2002). Flavonoids and arbutin from *Turnera diffusa*. Z Naturforsch.

[CR84] Piato AL, Detanico BC, Jesus JF, Rodrigues Lhullier FL, Nunes DS, Elisabetsky E (2008). Effects of Marapuama in the chronic mild stress model: further indication of antidepressant properties. J Ethnopharmacol.

[CR85] Plíšková M, Vondráček J, Křen V, Gažák R, Sedmera P, Walterová D, Psotová J, Šimánek V, Machala M (2005). Effects of silymarin flavonolignans and synthetic silybin derivatives on estrogen and aryl hydrocarbon receptor activation. Toxicology.

[CR86] Pockaj BA, Loprinzi CL, Sloan JA, Novotny PJ, Barton DL, Hagenmaier A, Zhang H, Lambert GH, Reeser KA, Wisbey JA (2004). Pilot evaluation of black cohosh for the treatment of hot flashes in women. Cancer Invest.

[CR87] Prasanna M (2000). Hypolipidemic effect of fenugreek: a clinical study. Indian J Pharmacol.

[CR88] Rafi MM, Rosen RT, Vassil A, Ho CT, Zhang H, Ghai G, Lambert G, DiPaola RS (2000). Modulation of bcl-2 and cytotoxicity of licochalcone-A, a novel estrogenic flavonoid. Anticancer Res.

[CR89] Rao SB, Chetana M, Devi PU (2005). *Centella asiatica* treatment during postnatal period enhances learning and memory in mice. Physiol Behav.

[CR90] Rhyu MR, Lu J, Webster DE, Fabricant DS, Farnsworth NR, Wang ZJ (2006). Black cohosh (*Actaea racemosa*, *Cimicifuga racemosa*) behaves as a mixed competitive ligand and partial agonist at the human μ opiate receptor. J Agric Food Chem.

[CR91] Rogerson S, Riches CJ, Jennings C, Weatherby RP, Meir RA, Marshall-Gradisnik SM (2007). The effect of five weeks of *Tribulus terrestris* supplementation on muscle strength and body composition during preseason training in elite rugby league players. J Strength Cond Res.

[CR92] Ruiz-Luna AC, Salazar S, Aspajo NJ, Rubio J, Gasco M, Gonzales GF (2005). *Lepidium meyenii* (Maca) increases litter size in normal adult female mice. Reprod Biol Endocrinol.

[CR93] Sautour M, Miyamoto T, Lacaille-Dibois MA (2006). Steroidal saponins and flavan-3-ol glycosides from *Dioscorea villosa*. Biochem System Ecol.

[CR94] Sautour M, Mitaine-Offer AC, Lacaille-Dubois MA (2007). The Dioscorea genus: a review of bioactive steroid saponins. J Nat Med.

[CR95] Schultes RE, Raffauf RF (1990). The Healing Forest.

[CR96] Seidlová-Wuttke D, Hesse O, Jarry H, Christoffel V, Spengler B, Becker T, Wuttke W (2003). Evidence for selective estrogen receptor modulator activity in a black cohosh (*Cimicifuga racemosa*) extract: comparison with estradiol-17β. Eur J Endocrinol.

[CR97] Seidlová-Wuttke D, Becker T, Christoffel V, Jarry H, Wuttke W (2003). Silymarin is a selective estrogen receptor β (ERβ) agonist and has estrogenic effects in the metaphysis of the femur but no or antiestrogenic effects in the uterus of ovariectomized (ovx) rats. J Steroid Biochem Mol Biol.

[CR98] Shao Y, Harris A, Wang M, Zhang H, Cordell GA, Bowman M, Lemmo E (2000). Triterpene glycosides from *Cimicifuga racemosa*. J Nat Prod.

[CR99] Shiau AK, Barstad D, Loria PM, Cheng L, Kushner PJ, Agard DA, Greene GL (1998). The structural basis of estrogen receptor/coactivator recognition and the antagonism of this interaction by tamoxifen. Cell.

[CR100] Simons R, Vincken JP, Mol LAM, The SAM, Bovee TFH, Luijendijk TJC, Verbruggen MA, Gruppen H (2011). Agonistic and antagonistic estrogens in licorice root (*Glycyrrhiza glabra*). Anal Bioanal Chem.

[CR101] Simons R, Gruppen H, Bovee TFH, Verbruggen MA, Vincken JP (2012). Prenylated isoflavonoids from plants as selective estrogen receptor modulators (phytoSERMs). Food Funct.

[CR102] Siqueira IR, Lara DR, Silva D, Gaieski FS, Nunes DS, Elisabetsky E (1998). Psychopharmacological properties of *Ptychopetalum olacoides* Bentham (Olacaceae). Pharmaceut Biol.

[CR103] Siqueira IR, Fochesatto C, da Silva AL, Nunes DS, Battastini AM, Netto CA, Elisabetsky E (2003). *Ptychopetalum olacoides*, a traditional Amazonian “nerve tonic”, possesses anticholinesterase activity. Pharmacol Biochem Behav.

[CR104] Spartan ’14 v.1.1.2 (2013) Wavefunction, Inc., Irvine, California, USA

[CR105] Spasov AA, Wikman GK, Mandrikov VB, Mironova IA, Neumoin VV (2000). A double-blind, placebo-controlled pilot study of the stimulating and adaptogenic effect of *Rhodiola rosea* SHR-5 extract on the fatigue of students caused by stress during an examination period with a repeated low-dose regimen. Phytomedicine.

[CR106] Sreeja S, Anju VS, Sreeja S (2010). *In-vitro* estrogenic activities of fenugreek *Trigonella foenum graecum* seeds. Indian J Med Res.

[CR107] Stone M, Ibarra A, Roller M, Zangara A, Stevenson E (2009). A pilot investigation into the effect of maca supplementation on physical activity and sexual desire in sportsmen. J Ethnopharmacol.

[CR108] Stonemetz D (2008). A review of the clinical efficacy of evening primrose. Holist Nurs Pract.

[CR109] Subathra M, Shila S, Devi MA, Panneerselvam C (2005). Emerging role of *Centella asiatica* in improving age-related neurological antioxidant status. Exptl Gerontol.

[CR110] Tamir S, Eizenberg M, Somjen D, Izrael S, Vaya J (2001). Estrogen-like activity of glabrene and other constituents isolated from licorice root. J Steroid Biochem Mol Biol.

[CR111] Tang W, Hioki H, Harada K, Kubo M, Fukuyama Y (2008). Clerodane diterpenoids with NGF-potentiating activity from *Ptychopetalum olacoides*. J Nat Prod.

[CR112] Tang W, Kubo M, Harada H, Hioki H, Fukuyama Y (2009). Novel NGF-potentiating diterpenoids from a Brazilian medicinal plant, *Ptychopetalum olacoides*. Bioorg Med Chem Lett.

[CR113] Tang W, Harada K, Kubo M, Hioki H, Fukuyama Y (2011). Eight new clerodane diterpenoids from the bark of *Ptychopetalum olacoides*. Nat Prod Commun.

[CR114] Thomsen R, Christensen MH (2006). MolDock: a new technique for high-accuracy molecular docking. J Med Chem.

[CR115] van Die MD, Burger HG, Teede HJ, Bone KM (2013). *Vitex agnus-castus* extracts for female reproductive disorders: a systematic review of clinical trials. Planta Med.

[CR116] Wang Y, Wang Y, McNeil B, Harvey LM (2007). Maca: an Andean crop with multi-pharmacological functions. Food Res Int.

[CR117] Waynberg J (1994). Yohimbine vs Muira puama in the treatment of sexual dysfunction. Am J Nat Med.

[CR118] Waynberg J, Brewer S (2000). Effects of Herbal vX on libido and sexual activity in premenopausal and postmenopausal women. Adv Ther.

[CR119] Wijeweera P, Arnason JT, Koszycki D, Merali Z (2006). Evaluation of anxiolytic properties of Gotukola – (*Centella asiatica*) extracts and asiaticoside in rat behavioral models. Phytomedicine.

[CR120] Wu G, Jiang S, Jiang F, Zhu D, Wu H, Jiang S (1996). Steroidal glycosides from *Tribulus terrestris*. Phytochemistry.

[CR121] Wuttke W, Gorkow C, Seidlová-Wuttke D (2006). Effects of black cohosh (*Cimicifuga racemosa*) on bone turnover, vaginal mucosa, and various blood parameters in postmenopausal women: a double-blind, placebo-controlled, and conjugated estrogens-controlled study. Menopause.

[CR122] Yan W, Ohtani K, Kasai R, Yamasaki K (1996). Steroidal saponins from fruits of *Tribulus terrestris*. Phytochemistry.

[CR123] Yuriev E, Ramsland PA (2013). Latest developments in molecular docking: 2010–2011 in review. J Mol Recog.

[CR124] Yuriev E, Agostino M, Ramsland PA (2011). Challenges and advances in computational docking: 2009 in review. J Mol Recog.

[CR125] Zakay-Rones Z, Varsano N, Zlotnik M, Manor O, Regev L, Schlesinger M, Mumcuoglu M (1995). Inhibition of several strains of influenza virus *in vitro* and reduction of symptoms by an elderberry extract (*Sambucus nigra* L.) during an outbreak of influenza B Panama. J Altern Complement Med.

[CR126] Zava DT, Dollbaum CM, Blen M (1998). Estrogen and progestin bioactivity of foods, herbs, and spices. Proc Soc Exp Biol Med.

[CR127] Zhang Y, Yu L, Ao M, Jin W (2006). Effect of ethanol extract of *Lepidium meyenii* Walp. on osteoporosis in ovariectomized rat. J Ethnopharmacol.

[CR128] Zhao J, Pawar RS, Ali Z, Khan IA (2007). Phytochemical investigation of *Turnera diffusa*. J Nat Prod.

[CR129] Zhao J, Dasmahapatra AK, Khan SI, Khan IA (2008). Anti-aromatase activity of the constituents from damiana (*Turnera diffusa*). J Ethnopharmacol.

[CR130] Zheng BL, He K, Kim CH, Rogers L, Shao Y, Huang ZY, Lu Y, Yan SJ, Qien LC, Zheng QY (2000). Effect of a lipidic extract from *Lepidium meyenii* on sexual behavior in mice and rats. Urology.

